# Comprehensive Investigation of Fluoroquinolone Residues in *Apis mellifera* and *Apis cerana* Honey and Potential Risks to Consumers: A Five-Year Study (2014–2018) in Zhejiang Province, China

**DOI:** 10.3390/toxics11090744

**Published:** 2023-08-31

**Authors:** Liang He, Leiding Shen, Jie Zhang, Rui Li

**Affiliations:** 1 Animal Experiment Center, Institute of Animal Husbandry and Veterinary Science, Zhejiang Academy of Agricultural Sciences, Hangzhou 310021, China; 2 Agricultural Economic Service Center, Jiaxing 314512, China; 3 Tongxiang Institute of Agricultural Sciences, Jiaxing Academy of Agricultural Sciences, Jiaxing 314512, China; 4 Agricultural Ministry Key Laboratory for Pesticide Residue Detection, Institute of Agro-Product Safety and Nutrition, Zhejiang Academy of Agricultural Sciences, Hangzhou 310021, China

**Keywords:** fluoroquinolones, residue, honey, risk assessment

## Abstract

As a group of antibiotics largely used in China’s animal husbandry, fluoroquinolone (FQ) residues in honey may pose potential threats to human health. This study performed a five-year investigation on the occurrence of FQ residues in honey in 521 *Apis mellifera* and 160 *Apis cerana* honey samples collected from Zhejiang Province, China and compared FQ residue profiles in honey with a subgroup of various factors. Deterministic and probabilistic risk assessments of exposure to FQ residues in honey were further conducted. Overall, four FQs were detected in 6.9% (47/681) of analyzed samples; banned norfloxacin with the highest level (7890 μg·kg^−1^) and detection frequency (4.9%) was the primary safety risk factor associated with honeybees raised in China. FQ detection frequency and concentration of rape honey was highest among four of the largest and most stable honeys (rape, acacia, chaste, and linden) in China. Processed honey from commercial sale channels had a significantly higher detection frequency of FQ residues than raw honey from apiaries. Deterministic assessment showed that the noncarcinogenic hazard quotient (HQ) value of the dietary intake of FQs by the local population was between 4.75 × 10^−6^ and 1.18 × 10^−3^, less than 1.0, indicating that FQ residues in honey posed a low risk for consumers. The order of the HQ value was ciprofloxacin > norfloxacin > enrofloxacin > ofloxacin. Probabilistic assessment showed that at P95, the HQ of FQs for the age groups of children, adolescents, adults, and older adults over 65 years ranged from 2.39 × 10^−5^ to 0.217, less than 1, and the exposure risk for adults was higher than for children and adolescents. Sensitivity analysis showed that FQ concentrations were the major contributors to health risks. Although a low risk was found, a strict hive management is needed for beekeepers regarding troubles of food safety, international trade, and human bacterial resistance.

## 1. Introduction

As a natural product, honey made by honeybees is appreciated by consumers worldwide for its nutritional properties. China is the largest global supplier of honey; its honey consumption exceeds 300,000 tons per year [1,2]. Like all living organisms, honeybees can be infected with bacterial pathogens, such as European foulbrood and American foulbrood [2]. Some beekeepers illegally use antibiotics for preventing and treating honeybee diseases [3]. An accumulation of antibiotic residues in honey usually results from the overuse of antibiotics, causing poor product quality and trade difficulties [4]. Residual antibiotics directly cause safety issues, such as toxic effects, allergenic potential, and increased bacterial resistance [5]. As a result, antibiotic residues in honey have been widely studied with regard to public health in recent years.

Antibiotics often applied on honeybees include enrofloxacin, lincomycin, tetracycline, streptomycin, sulfonamide, etc. However, in European countries, the United States (USA), Canada, and China, the use of these agents in beekeeping is strictly regulated or illegal due to the lack of tolerance for antibiotic residues in honey [2]. Fluoroquinolones (FQs) are the most widely applicated antibiotics in China, accounting for nearly 15% of the total antibiotic consumption in humans and animals [6]. FQs comprise a group of broad-spectrum antibiotics, such as ciprofloxacin, danofloxacin, enrofloxacin, norfloxacin, ofloxacin, pefloxacin, etc. [5]. By 2015, the use of enrofloxacin was banned in food animals in the USA [7]. Ciprofloxacin was banned for use in aquaculture by the European Union (EU) and North America [8]. The Chinese government issued notice 2292, banning the use of four FQs (norfloxacin, ofloxacin, lomefloxacin, and pefloxacin) for all edible animals [9]. At present, FQs’ efficacy for controlling honeybee diseases is still controversial; however, the use of them in apiculture has obviously risen in recent years, especially in Asia [2]. Between 2006 and 2007, ciprofloxacin, norfloxacin, and enrofloxacin were found in about 75.3% of honey samples in retail outlets in Switzerland, with concentrations ranging from 1.4 to 225.0 μg·kg^−1^ [10]. Another survey found that the highest concentrations of norfloxacin, ciprofloxacin, ofloxacin, and enrofloxacin were 397.1, 74.2, 29.7, and 281.4 μg·kg^−1^, respectively, and the detection rate of norfloxacin was 5.4% in bee products from Chinese apiaries and supermarkets in 2014 [11]. The survey from Wang et al. also showed that quinolones were found in 69.1% of 96 honey samples from the biggest online sale platform in China: Taobao [12]. To date, no maximum residue limit (MRL) has been set by EU for residual antibiotics in honey, which also indicates that any residual antibiotics of honey is unallowed. 

Western honeybees (*Apis mellifera*) and Chinese honeybees (*Apis cerana*) are cultivated with different management practices in China to produce honey [13]. *Apis mellifera* are more suitable for modern breeding and are more productive. In order to achieve greater economic benefit, Chinese beekeepers prefer to produce uncapped immature *Apis mellifera* honey to improve honey yield by repeated extraction [14]. The immature honey ferments easily with reduced quality and, thus, Chinese enterprises need to preheat and concentrate available immature honey to temperatures exceeding 60 °C to meet the legal food criteria [15]. Compared with *Apis mellifera*, *Apis cerana* have not been as intensively bred and selected; each colony produces no more than 20 kg of honey a year [16]. In fact, *Apis cerana* beekeepers mainly produce naturally mature honey for high prices, good quality, and consumer preference in China. In published studies, the occurrence of antibiotic residues and risk assessments mainly focused on *Apis mellifera* honey from markets [10,17]. However, there is limited information on differences in antibiotic residual levels in honey from *Apis mellifera* and *Apis cerana*. In China, direct purchases from farmers are becoming increasingly common for consumers, notably in terms of primary agro-products with typical regional characteristics, which may pose a higher threat to food safety. At present, there are four main kinds of offline sale channels that sell honey: supermarkets, honey processing facilities, bee product stores, and apiaries. As far as we know, there is almost no information available on comparing the occurrence of antibiotic residues in honey from various sale channels and on dietary risk assessments of antibiotics in honey.

As a major honey-producing region, East China’s Zhejiang Province has over one million honeybee hives, with approximately 15,000 beekeepers, accounting for one-seventh of the national output [18]. Nevertheless, large-scale investigations of FQ residue profiles in honeys from Zhejiang Province are limited. Consequently, the object of this study was to comprehensively investigate FQ residues in *Apis mellifera* and *Apis cerana* honey from various floral origins in four types of offline sale channels (apiaries, supermarkets, honey processing facilities, and bee product stores), covering the major honey-producing regions of Zhejiang Province in China. Finally, deterministic and probabilistic risk assessment methods were used to evaluate potential health risks to local residents following dietary exposure to residual FQs in honey.

## 2. Materials and Methods

### 2.1. Sampling

**Figure 1:**
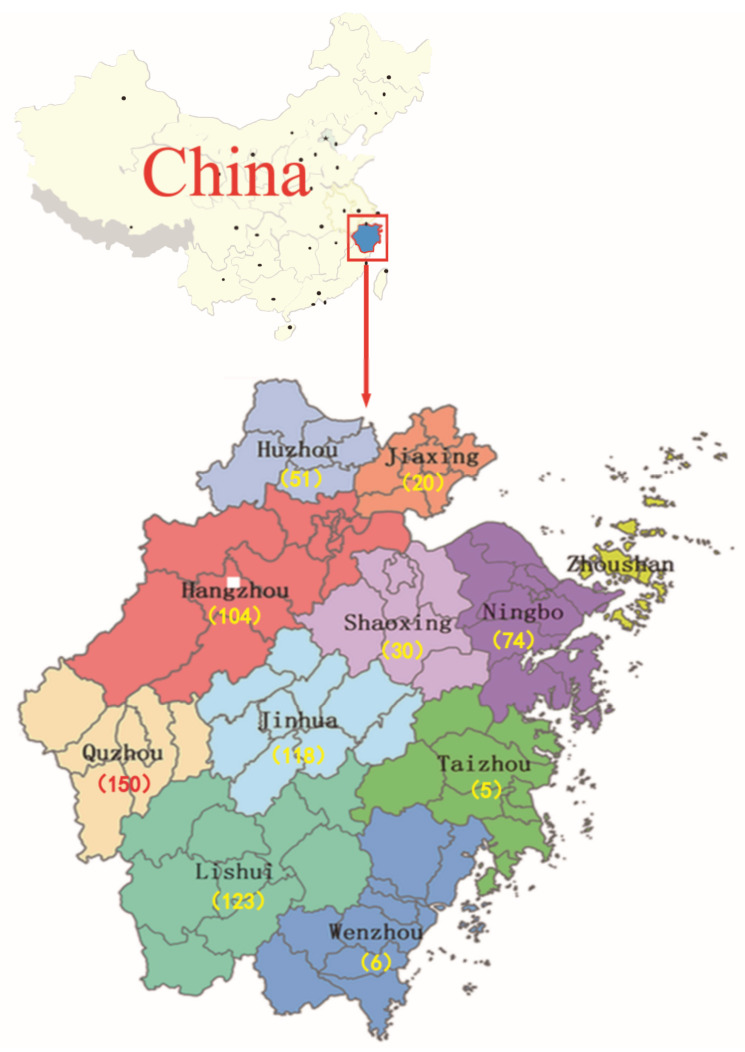
Location and number of honey samples collected from Zhejiang Province in China.

There was a total of 681 samples (521 *Apis mellifera* honey and 160 *Apis cerana* honey) collected randomly from 2014 to 2018, covering the major honey-producing regions of Zhejiang Province, China. The locations and numbers of samples collected are shown in Figure 1. The number of samples was 82, 153, 115, 189, and 142 in 2014, 2015, 2016, 2017, and 2018, respectively. Samples were from 502 apiaries, 70 supermarkets, 67 honey processing facilities, and 42 bee product stores, and originated from 26 floral plants, covering most of the floral plants in China, as shown in Supplementary Materials (Table S1). Information regarding the sampling strategy in detail, including sampling channel, location, and year of each sample, is provided in the Supplementary Materials (Table S2). Each sample of honey was at least 250 g. Samples were immediately stored in a cooler with ice bags at 4 °C and detected within a week. Crystallized honey was dissolved to ensure its homogeneity by water bath heating at 40 °C before analysis. 

### 2.2. Reagents and Chemicals

**Figure 2:**
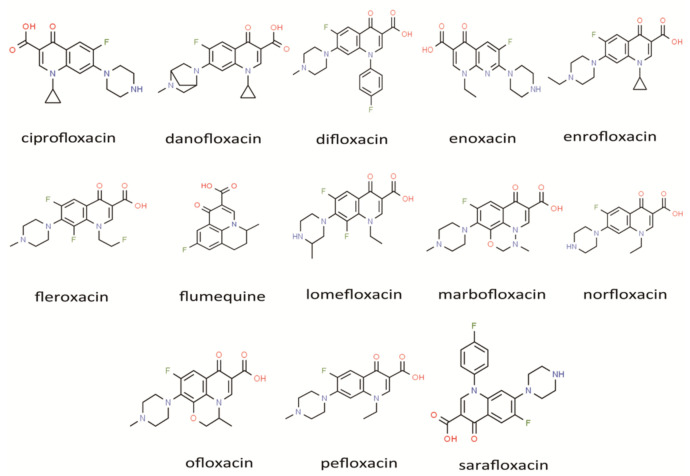
The structure of targeted fluoroquinolones.

All FQ standards (ciprofloxacin, danofloxacin, difloxacin, enoxacin, enrofloxacin, fleroxacin, flumequine, lomefloxacin, marbofloxacin, norfloxacin, ofloxacin, pefloxacin, and sarafloxacin) with a purity degree above 98.0% were supplied by Dr. Ehrenstorfer GmbH Co. (Augsburg, Germany); the structure of all FQs is shown in Figure 2. FQ standards were diluted by methanol at a concentration of 100 µg/mL as standard stock solutions. The required concentrations of standard solutions were prepared by appropriately diluting the stock solution and then stored in a refrigerator at 4 °C. HPLC-grade acetonitrile, formic acid, and methanol were obtained from Tedia Ltd. Analytical-grade ammonium formate was supplied by Shanghai Topscience Biotechnology Co., Ltd. Oasis PRiME HLB (200 mg, 6 cc, Waters Corporation, Milford, MA, USA) was purchased from Hangzhou Rongxinherun Biotechnology Co., Ltd. Ultrapure water was prepared by using the Millipore system (Molsheim, France).

### 2.3. Apparatus and LC-MS/MS Conditions

LC-MS/MS analysis was performed on an LC-30AD ultraperformance liquid chromatograph (Shimadzu, Japan) coupled with a QTRAP 6500 mass spectrometer (Applied Biosystems, Waltham, MA, USA). Chromatographic column (Waters ACQUITY BEH C18, 2.1 mm × 100 mm, 1.7 µm) was used for separating the FQs in honey samples. The mobile phases, consisting of A (methanol) and B (2 mmol/L ammonium formate containing 0.1% formic acid), were set at the flow rate of 0.3 mL/min. The elution gradient was as follows: 0–1.0 min (B: 95%), 1–3 min (B: 95–80%), 3–7.5 min (B: 80–5%), 7.5–10 min (B: 5%), 10–10.1 min (B: 5–95%), and 10.1–12.0 min (B: 95%). The injection volume of the sample extract was 5 µL. Source temperature was 500 °C and ion spray voltage was set at 4.5 kV; curtain gas, gas 1, and gas 2 pressures were set at 40 psi, 55 psi, and 50 psi, respectively. The MRM (multiple reaction monitoring) transition parameters are listed in the Supplementary Materials (Table S3). 

### 2.4. Extraction Process 

The extraction and cleaning of FQs from honey samples was performed according to our previously published method with minor modifications [19]. Briefly, honey (1.0 g) was added to 4 mL acetonitrile and 1 mL sterile water in a 15-mL conical plastic tube, then vortexed to be entirely dissolved. The homogenized mixture was centrifuged at 12,000× *g* for 180 s. Then, 2.5 mL of supernatant extract was transferred into the Oasis PRiME HLB (200 mg/6 mL, Waters), with a combination of novel solid phase extraction (SPE) adsorbent materials in one cartridge with the main advantage of simplifying and accelerating the SPE process. The sample portion was allowed to gravity drain through the cartridge to collect the eluates. Finally, each eluate was filtered through a 0.22 μm nylon membrane to be injected into the LC-MS/MS. 

### 2.5. Quality Assurance/Quality Control

Strict measures of quality assurance and control were based on the realization of blank samples covering all the analytical phases. During the whole sequence, quality control samples were injected periodically to ensure the system stability and analysis accuracy. For each batch of 30 samples, 2 uncontaminated blank honey samples spiked at 0.005 mg/kg and 0.05 mg/kg of FQs were used to check the accuracy of sample analysis (recoveries ranged between 70% and 120%). Five working solutions of honey matrix-matched standards (0.1–200 μg/L) were used, with the linear regression coefficients of R^2^ for all the FQs. 

### 2.6. Assessment of Dietary Risk to Consumers

The European Food Safety Authority presented the EDI (estimated daily intake) and ADI (acceptable daily intake) to assess health risks. The hazard quotient (HQ) of ingesting the residual FQs in honey was calculated as follows: HQ = EDI/ADI, where ADIs of FQs were obtained from authorities (Chinese National Standard) or the literature [20,21,22]. The exposure risk was considered acceptable when the HQ value was lower than 1.

#### 2.6.1. Deterministic Risk Assessment

A deterministic method was used to calculate the EDI covering the average daily exposure over the entire lifetime [23]. WHO-recommended methods were used to calculate the EDI and hazard quotient (HQ) to evaluate the chronic exposure risks to consumers [24]. The EDI_d_ was calculated as follows: EDI_d_ = (C_d_ × K_d_)/BW_d_, where C_d_ (μg·kg^−1^) is each compound residue average concentration, K_d_ is the average daily consumption of honey (0.01 kg/day), and body weight (BW_d_) for adults is adopted as 60 kg in China [25]. 

#### 2.6.2. Probabilistic Risk Assessment

The Monte Carlo simulation was used for the probabilistic assessment of pollutant exposure to describe the uncertainty that was pervasive in the risk assessment as a result of the lack of precise knowledge and the variability of individual human characteristics [26]. Based on the Monte Carlo simulation, probabilistic risks were evaluated by Crystal Ball (version 11.1.2.4.400 (32-bit) Oracle, Inc., Austin, TX, USA). The number of replications for each equation was at least 10,000 and the values at different quantiles (50th and 95th) of the exposure distribution were used to assess the probabilistic risk with considering triangular distribution for residue concentration and lognormal distribution for ingestion rate and body weight, after fitting analysis in the Monte Carlo simulation [27,28]. EDI_j_ was computed as follows: EDI_j_ = (C_j_ × K_j_)/BW_j_, where C_j_ (μg·kg^−1^) is each FQ’s residue concentration; K_j_ is daily honey intake (kg/day); K_j_ and BW_j_ (body weight, kg) are surveyed based on the 24 h recall method. In this survey, 1956 inhabitants from 4 groups of representative populations were randomly selected in Zhejiang Province, including children aged 4–11 years, adolescents aged 12–18 years, adults aged 19–64 years, and older adults over 65 years old in 2019. The means and standard deviations of FQ exposure parameters (C_j_, K_j_, and BW_j_) for various age groups are shown in the Supplementary Materials (Table S4).

### 2.7. Statistical Analysis

Samples with FQ residues at least more than the limits of quantitation (LOQ) were considered positive. Data analyses were performed using SPSS software v17.0 and Microsoft Excel v2010. Statistical analysis data were expressed as the mean ± standard deviation. As residue concentrations in honey did not follow a normal distribution, the non-parametric Kruskal–Wallis ANOVA test and Chi-square test were respectively used to evaluate the differences of FQ residue levels and detection frequencies in honey samples; the differences were considered statistically significant when *p* < 0.05. According to the guideline of Scientific Cooperation Task 3.2.10 of the European Commission (SCOOP), non-detection was expressed as half the limit of detection (LOD/2) of the FQ residue during the dietary risk assessment. 

## 3. Results

### 3.1. Validation of the Analytical Methodology

Matrix-matched calibration standards by means of the external calibration method were used in our study. Validated parameters (retention time, linearity, limits of detection (LOD), LOQ, and matrix effects) of all compounds are shown in Table 1. LODs and LOQs were 0.00015–0.001 and 0.00053–0.0031 mg·kg^−1^ in multiple ion-monitoring modes, respectively. Good linearity results with regression coefficients greater than 0.99 were obtained for all FQs. The matrix effect (ME) was –20% to 20% for 13 target compounds, indicating acceptable ME for residue analysis. Blank honey samples were added to target analytes at three concentrations (0.005, 0.025, and 0.05 mg·kg^−1^); recoveries ranged from 77.3 ± 6.2% to 110.8 ± 5.8%, where the coefficient of variation was lower than or equal to 15%. 

### 3.2. FQ Overall Detection Rate and Concentration in Honey 

**Figure 3:**
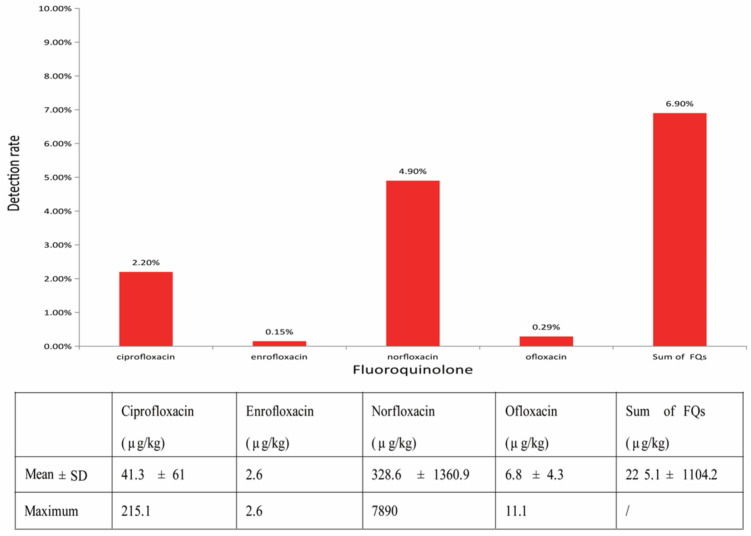
FQs detection rates in all honey samples and FQs residue levels in positive samples.

A total of 4 FQs (ciprofloxacin, enrofloxacin, norfloxacin, and ofloxacin) above the LOQ of the method were detected in 6.9% (47/681) of all analyzed samples, whereas the other 9 FQs were not detected. Among all positive samples, 93.6% contained a single FQ, 6.4% contained two or three of the FQs, and none of the samples contained over three target compounds. The highest FQ concentration detected among positive samples was 7890 μg·kg^−1^; the mean concentration was 225.1 ± 1104.2 μg·kg^−1^ (Figure 3). In general, among positive samples, two sample concentrations were over 1000 μg·kg^−1^; 12.7% were between 100 and 1000 μg·kg^−1^; 44.7% were between 10 and 100 μg·kg^−1^; 36.2% were below 10 μg·kg^−1^. FQ residue concentrations in 2.5% of all honey samples did not comply with the uniform limit (10.0 μg·kg^−1^) in the EU and Japan. 

### 3.3. Each FQ Detection Rate and Concentration in Honey 

The largest detection rate was for norfloxacin (4.9%), followed by ciprofloxacin (2.2%), ofloxacin (0.29%), and enrofloxacin (0.15%) in all samples (Figure 3). The mean concentration of norfloxacin detected among positive samples was 328.6 ± 1360.9 μg·kg^−1^. The concentrations of norfloxacin in 4.3% of positive samples were in the range of over 1000 μg·kg^−1^; 8.5% were between 100 and 1000 μg·kg^−1^; 38.3% were between 10 and 100 μg·kg^−1^; 19.2% were below 10 μg·kg^−1^. Our results show that norfloxacin in honey is the most important safety risk factor associated with honeybees raised in China. The mean concentration of ciprofloxacin was 41.3 ± 61 μg·kg^−1^. Concentrations of ciprofloxacin in 4.3% of positive samples were between 100 and 1000 μg·kg^−1^; 10.9% were between 10 and 100 μg·kg^−1^; 17.4% were below 10 μg·kg^−1^. The proportion of ofloxacin positive samples with concentrations (10–100 μg·kg^−1^) was 2.2%, with the mean concentration of 6.8 ± 4.3 μg·kg^−1^; the proportion of positive samples with concentrations below 10 μg/kg was also 2.2%. Only one sample containing enrofloxacin had a concentration (2.6 μg·kg^−1^) below 10 μg·kg^−1^, with 0.15% of the detection rate (Figure 3). 

### 3.4. Comparison of FQ Detection Rate and Concentration in Honey among Different Areas, Years, Entomological Origins, Floral Origins, and Sale Channels

Detection rate and residue level varied considerably among the regions of Zhejiang Province. The top three cities with the highest detection frequency of FQs were Ningbo city (17.6%), followed by Huzhou city (11.8%) and Shaoxing city (10.0%), mainly from plain areas. These values were higher than those from Jinhua City (3.4%) and Lishui City (3.3%), mainly from mountain areas (*p* < 0.05). FQ mean concentrations of five cities (Quzhou, Hangzhou, Lishui, Huzhou, and Ningbo) were at the highest levels in Zhejiang Province. FQ mean concentrations in the positive samples from these five cities were between 41.1 ± 49.9 and 757.7 ± 2180.5 μg·kg^−1^, which was far higher than the 10×LODs in this study (Table S5). The average FQ concentrations of positive samples in the remaining five cities ranged from 11.3 ± 14.0 to 30.9 ± 30.5 μg·kg^−1^. The detection rates and concentrations varied considerably from 2014 to 2018. The detection rate of FQs was the highest in 2016 (13.0%), followed by 2018 (9.9%), 2014 (6.1%), 2017 (5.8%), and 2015 (1.3%). The average FQ residue levels of all positive samples from 2014 to 2018 were in the range of 15.5 ± 18.2–773.0 ± 2362.7 μg·kg^−1^ (Table S6). 

In total, the FQ detection frequency was 7.9% of honey samples from *Apis mellifera*, which was significantly higher than (3.8%) from *Apis cerana* (*p* < 0.05). Mean concentration (251.5 ± 1172.9 μg·kg^−1^) of positive *Apis mellifera* honey was also significantly higher than (27.0 ± 28.2 μg·kg^−1^) of positive *Apis cerana* honey (*p* < 0.05). Only one FQ was found in all positive *Apis cerana* honey samples, whereas 92.5% of positive samples from *Apis mellifera* contained one FQ and 7.5% contained two or three FQs. In addition, only norfloxacin and ciprofloxacin were found in honey from *Apis cerana,* with the detection rates of 2.5% and 1.25%, respectively. Norfloxacin was found in 5.6% of *Apis mellifera* honey, followed by ciprofloxacin (2.5%), ofloxacin (0.38%), and enrofloxacin (0.19%). FQs were found in nine types of floral plants, including the main plants used producing commercial honey such as acacia, rape, and citrus. The floral origin with the highest detection frequency of FQ residues was motherwort (100%), followed by Chinese milk vetch (60%), honeysuckle (50.0%), jujube (10%), rape (7.4%), loquat (6.1%), citrus (5.9%), acacia (5.8%), and mutiflower (5.7%). The floral plants with the highest FQ mean concentrations detected in positive samples were rape (424.7 ± 1754.7 μg·kg^−1^), followed by mutiflower (158.4 ± 101.1 μg·kg^−1^), Chinese milk vetch (124.8 ± 89.2 μg·kg^−1^), acacia (65.3 ± 61.8 μg·kg^−1^), loquat (39.3 ± 26 μg·kg^−1^), honeysuckle (31.3 μg·kg^−1^), motherwort (16.2 ± 11.9 μg·kg^−1^), citrus (8.1 ± 0.18 μg·kg^−1^), and jujube (5.7 μg·kg^−1^). From our results, the quality safety of honey from rare special floral plants should be given the strictest attention, such as motherwort, Chinese milk vetch, and honeysuckle. 

Chinese consumers purchase honey mainly from four offline sale channels (apiaries, supermarkets, honey processing facilities, and bee product stores). The sale channel with the highest detection frequency of FQ residues was honey processing facilities (14.9%), followed by supermarkets (12.9%), bee product stores (9.5%), and apiaries (4.8%).The highest mean FQ concentration detected in positive samples was from bee product stores (376.8 ± 640.6 μg·kg^−1^), followed by apiaries (310 ± 1054.1 μg·kg^−1^), supermarkets (89.2 ± 109.7 μg·kg^−1^), and honey processing facilities (48.8 ± 71.9 μg·kg^−1^).

### 3.5. Assessment of Dietary Exposure Risk to Humans

The deterministic assessment of exposure to risky FQs through ingesting honey among consumers is shown in Table 2. The EDIs of FQs were between 1.52 × 10^−5^ and 2.68 × 10^−3^ μg·kg^−1^ bw·day^−1^; HQs were between 4.75 × 10^−6^ and 1.18 × 10^−3^. The order of the noncarcinogenic risk was the HQ of ciprofloxacin (1.18 × 10^−3^) > norfloxacin (1.91 × 10^−4^) > enrofloxacin (6.45 × 10^−6^) > ofloxacin (4.75 × 10^−6^). HQs of FQs in honey were less than 1, indicating acceptable risk levels. Even when using the worst case scenario approach (highest concentration found), EDI values were up to 9.39% of the ADI of norfloxacin found in this study, also meaning acceptable risk levels. The EDIs and HQs of FQ residues in Western and Chinese honeybees honey were from 1.25 × 10^−5^ to 3.92 × 10^−3^ μg·kg^−1^ bw·day^−1^ and 3.91 × 10^−6^ to 1.46 × 10^−3^, respectively (Table 3). The results further indicated that the potential risks of FQ residues in honey from Western honeybees were higher than those of Chinese honeybees for consumers. The EDIs and HQs of FQ residues in various floral origins of honey were from 1.39 × 10^−5^ to 1.24 × 10^−2^ μg·kg^−1^ bw·day^−1^ and 2.09 × 10^−6^ to 8.27 × 10^−2^, respectively (Table 4). The results also showed that the potential risks of FQ residues in Chinese milk vetch honey were the highest for consumers, followed by rape honey and honeysuckle flower honey. The dietary exposure assessment of risky FQs detected in various sale channels showed that the EDIs and HQs were between 1.70 × 10^−5^ and 5.85 × 10^−3^ μg·kg^−1^ bw·day^−1^ and 5.3 × 10^−6^ and 1.29 × 10^−3^, respectively, indicating that the potential risks of FQ residues in supermarket were the highest for consumers compared with those of the other three sale channels (Table 5). The potential risks of FQ residues in Chinese milk vetch honey were the highest for consumers, followed by loquat and rape honey (Table 5). 

**Figure 4:**
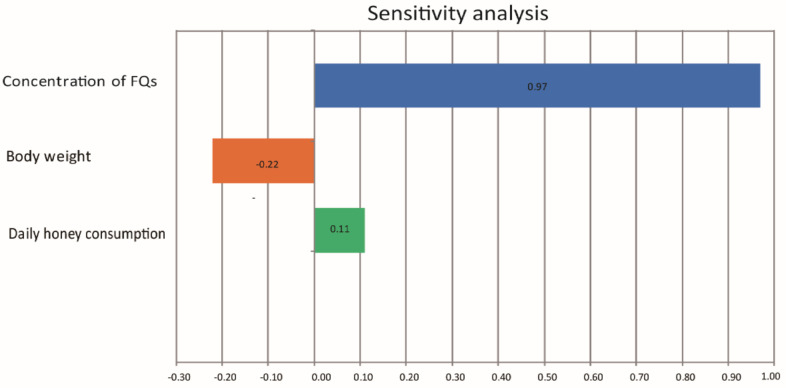
The influence of different parameters on the calculated HQs.

The probabilistic assessment of exposure to risky FQs through ingesting honey among consumers is shown in Table 6. This study showed that at P95, the HQs of ciprofloxacin for the age groups of children (4–11 years), adolescents (12–18 years), adults (19–64 years), and older adults over 65 years reached 7.87 × 10^−2^, 0.102, 0.217, and 0.199, respectively. At P95, the HQ of enrofloxacin for the age groups of children (4–11 years), adolescents (12–18 years), adults (19–64 years), and older adults over 65 reached 2.39 × 10^−5^, 3.08 × 10^−5^, 5.92 × 10^−5^, and 6.0 × 10^−5^, respectively. At P95, the HQ of norfloxacin for the age groups of children (4–11years), adolescents (12–18 years), adults (19–64 years), and older adults over 65 reached 2.94 × 10^−2^, 4.01 × 10^−2^, 8.57 × 10^−2^, and 7.86 × 10^−2^, respectively. At P95, the HQs of ofloxacin for the age groups of children (4–11 years), adolescents (12–18 years), adults (19–64 years), and older adults over 65 reached 1.94 × 10^−4^, 2.50 × 10^−4^, 5.38 × 10^−4^, and 4.88 × 10^−4^, respectively. The dietary exposure levels of FQ levels for adults (19–64 years) and older adults over 65 were high among all age groups. In summary, the dietary risk from these FQs for consumers in all age groups was acceptable (HQ < 1). Based on the Monte Carlo simulation sensitivity analysis, in the case of HQ for FQs, FQ concentration had the greatest impact on the risk of exposure. Body weight had the lowest effect on the calculated HQ for FQs in honey (Figure 4).

## 4. Discussion

As a complex natural agro-product, honey contains glucose, fructose, vitamins, organic acids, minerals, etc. [29]. The residual antibiotic concentrations of honey are usually at trace levels; thus, accurate analysis of antibiotic residue requires the use of LC-MS/MS with high selectivity and sensitivity. Some analytical methods for determination of FQ residues in honey have been reported, including the Chinese National Standard GB 31657.2-2021 [11]. The Oasis PRiME HLB cartridge has been developed recently as a pass-through type SPE cartridge, which is effective for rapid pass-through cleanup of various food matrices in food safety analyses. Compared with general SPE cartridge (Oasis HLB and Strata-X cartridges) and QuEChERS cleanup, Oasis PRiME HLB cartridges have no requirement for conditioning or equilibration prior to use and significantly reduce sample preparation time [30]. Our validation results indicate that this modified PRiME methodology is effective and reliable for FQ residue determination in honey. 

The findings of this study were further compared to some of the data presented in other studies regarding the occurrence of FQ residues in honey. The EU data showed the presence of quinolones (3%) in honeybee products from 2009 to 2013 [31]. FQs were not detected in 74 honey samples from the Italian market containing different botanical origins and geographical places, 20 commercial honey samples from Cyprus and Greece of different types, and 110 locally produced European Flemish honey from 2006 to 2015 [32,33]. FQ residues were detected in 6.7% of honey samples purchased from Japan [34]. In our study, the mean concentration (225.1 μg·kg^−1^) of FQs detected among positive samples was significantly higher than (7.3 μg·kg^−1^) honey samples collected from different regions of southern Italy in 2018–2019 [35] Overall, the detection frequency and residue levels of FQ residues in Chinese honey was generally higher than that of developed countries; the reason might be the over-use of antibiotics in China due to low honey safety awareness and weak antibiotic management in beekeeping [36]. Meanwhile, sampling areas, sample sizes, or analytical sensitivity may affect this conclusion in these studies. 

Our results indicated that norfloxacin with the highest detection frequency was the important safety risk factor associated with honeybees raised in China, being in accordance with the detection frequency of norfloxacin (5.4%) from one Chinese survey in 2017 and the detection frequency of norfloxacin (42.6%) from another Chinese study in 2022 (42.6%) [11,12]. In this survey, the detection frequency of ciprofloxacin was 2.2%, which was lower than the 12.8% of a previous study within all 94 honey samples from the Taobao website and 4.4% in 46 honey samples from honey producers and cooperatives located in the cities of Northern China [12,37]. In honey from Chinese Zhejiang Province, the detection frequency of enrofloxacin was 0.15%, far below the 20.7% of 135 honey samples in the range of 0.6–72.1 μg·kg^−1^ collected randomly from Iranian Qazvin Province and 2.5% of 80 commercial blossom honey samples obtained from local markets in Ankara, Turkey [38,39].

We also found that the detection rates and concentrations of FQs in honey from Chinese Zhejiang Province varied considerably among the regions and years. Different FQ residue levels in honey among various regions might be attributed to different drug utilization in two types of entomological origins (*Apis mellifera* and *Apis cerana*) and specific geographical conditions (plain and mountain areas). Due to more honey production and commercial benefits, a majority of professional beekeepers choose to raise *Apis mellifera* in China. Small amounts of Chinese beekeepers and hobbyists, especially in mountain areas, choose to raise indigenous Chinese honeybees with less management and high quality [18]. Our results showed that the detection frequency and residue levels of FQs in honey from Western honeybees were obviously higher than Chinese honeybees (*p* < 0.05), which may be related to the fact that Chinese honeybee colonies live in mountainous regions, consume natural nectar, and have little exposure to veterinary drugs. Meanwhile, Chinese honeybees are more resistant to disease than Western honeybees and typically produce mature honey 1–2 times a year by cutting the honeycomb with high prices, which may result in less antibiotic use. Moreover, since Chinese honeybees are resistant to the major bee pest, this may also enhance bees’ health levels and reduce drug usage [18]. The different detection rates of FQs in various years may be linked to climate change and the outbreak of honeybee disease in China between 2016 and 2018. Thus, more caution should be taken regarding antibiotic control in a particular year when honeybee disease outbreaks occur.

Approximately 40 types of floral plants produce honey in China; rape, acacia, chaste, and linden honeys are the four largest and most stable honeys, with over 90% of Chinese honey gross output, especially rape honey, which accounts for nearly 50% [40]. FQ detection rate and concentrations of rape honey were highest among four honeys (rape, acacia, chaste, and linden). Though there was no statistically significant difference between rape honey and other honey (acacia, chaste, or linden), the quality safety of rape honey should be firstly concerned. The most important reason may be related to rape being the first major food source for honeybees after winter. Chinese beekeepers often use antibiotics to prevent various honeybee diseases during the buildup of bee colonies just coming out of winter. Another important reason may be related to the lower price of rape honey. Beekeepers usually use more antibiotics in adverse circumstances to keep honeybees healthy, leading to the production of cheaper honey [41]. 

Regarding FQ residues, our results showed that the quality of honey from traditional offline sale channels with low FQ detection frequency appeared to be better than that of honey from online electronic businesses, a finding that was supported by a survey that the detection frequency of quinolone was 69.1% in honey from the Taobao website [12]. FQ detection frequency in apiaries was the lowest among four sale channels, in according with the detection frequency (4.8%) in honey samples from apiaries of honey producers and cooperatives in Northern China [37]. In China, most commercial honey products from supermarkets and honey processing facilities are processed from immature harvest honey with cheaper prices and lower production costs [42]. In this study, FQ detection frequency in processed honey from commercial sale channels was significantly higher than in raw honey from apiaries (*p* < 0.05), which might be caused by a mixture of small amounts of highly contaminated honey in vacuum dryers during honey processing. Thus, the quality of commercial honey should be improved to meet the demands of consumers in China. Control of antibiotic residues in raw honey from apiaries before processing is a major challenge in the honeybee product industry in China.

Both deterministic and probabilistic methods can be used for risk assessment of exposure to pollutants. Based on single-point estimates, the deterministic method is relatively easy to carry out and the single-risk estimate output is easy to understand and interpret [43]. The probabilistic method combines chemical occurrence information and dietary data to estimate the distribution of intakes within a population and achieves a more realistic estimate of exposure by incorporating variability and uncertainty into the assessment [43]. Until now, there has been no convincing evidence that FQs have carcinogenic effects [44]. Thus, noncarcinogenic health risk assessments were performed without considering a threshold carcinogenic risk in our study. 

To our knowledge, just a few investigations have been conducted on human dietary exposure to antibiotic residues in honey. The exposure risk to human health from common quinolone residues (norfloxacin, ciprofloxacin, enrofloxacin, ofloxacin, and marbofloxacin) in 96 honey samples collected from the Chinese market were evaluated by using the deterministic method; the HQs (1.71 × 10^−5^–1.80 × 10^−2^) of these quinolones were all less than 1.0, meaning a low health risk for exposed consumers. However, the sample size was relatively small, resulting in uncertainty of this result [12]. Another deterministic exposure assessment showed that HQs of chlortetracycline residue in honey from South Korea were 1.58 and 3.22 for children and adults, respectively, and HQs of other antibiotics (macrolide, lincosamide, sulfonamide, aminoglycoside, and penicillin) were less than 1.0. These results suggested that the risk of tetracycline from honey consumption was the highest for human health [45]. For honey collected from Nigeria’s Adamawa state, HQs estimated for the individual antibiotics (tetracycline, streptomycin, sulfonamide, and chloramphenicol) were observed to be <1.0, indicating that the potential risk arising from consumers exposed to different antibiotic residues through the consumption of the honey fell below the level of concern [46]. Our results of the deterministic exposure assessment also showed that all EDI values were lower than the ADI of FQs, meaning that the health risk of FQ residues in honey was low for human health. Deterministic exposure assessment further indicated that the potential risks of FQ residues in honey from supermarkets and Chinese vetch honey were higher than those from other sale channels and floral origins, respectively.

By combining dietary data and pollutant occurrence information, probabilistic methods based on the Monte Carlo simulation estimated the intake distribution among individuals within a population and achieved a more realistic assessment of exposure compared with the deterministic methods by incorporating variability and uncertainty into the assessment [47]. Results from the probabilistic exposure assessment of pesticide (chlorpyrifos and cypermethrin) residues in vegetables and veterinary drug (leucomalachite green) residues in fish products indicated that children (2–6 years) and adolescents (13–18 years) were considered the most vulnerable group [43,48]. To the best of our knowledge, this is the first time that the daily intake of FQs in honey was probabilistically assessed worldwide. Honey is strictly forbidden to be given to children under 1 year old and not recommended for children under 3 years old in China. Thus, our dietary survey excluded young children (<4 years). Our probabilistic dietary risk results indicated that the exposure risk for adults was higher than that of children (4–11 years) and adolescents (12–18 years), which might be explained by the Chinese young population rarely eating honey due to more commercial soft drink substitutes or traditional honey diet culture loss. 

In the dietary exposure assessments of some studies, uncertainty analysis was an important content to correctly understand the limits and strength of its results [49,50]. Although we performed the dietary risk assessment based on 681 samples obtained from a 5 year survey by using both deterministic and probabilistic approaches in this study, uncertainties were still unavoidably involved in the process of the present work, arising from insufficient knowledge about exposure scenarios, models, and inputs to the models. The first uncertainty was from dietary exposure; we did not consider the total dietary risk for FQs for lack of data, such as consumption data, or FQ concentrations data in other food commodities (milk, eggs, fish, etc.). In addition, FQ concentration was directly detected from raw samples collected from apiaries, without considering potential changes in FQ concentration of honey during its processing and cooking. If processing factors were applied, the estimated exposure would be lower. Secondly, there might be an uncertainty of the adopted ADIs. Because extremely low levels of antibiotics might also alter the human microbiome, the health risk assessment based on current ADIs could be conservative [51]. Considering that adopted ADIs values are established only on the basis of the toxicological effects of FQs and microbiological minimum inhibitory concentration, there is still some uncertainty in extrapolating results from animal experiments to humans. The third uncertainty was measurement uncertainty; non-detects should be set to the lower and upper bound scenarios, representing the variation between an optimistic and worst case scenario. However, a worst case scenario depended on the actual LOD and LOQ of the analytical method in this study without guarantying that the result would be optimistic or pessimistic. Furthermore, the special sensitive groups (such as pregnant or lactating women or people with liver and kidney functional disorder) susceptible to the drugs were not involved due to lack of relevant data. Moreover, the model used for exposure assessment was closely related to uncertainty analysis. Deterministic models are generally used as a first step or screening assessment without requiring any uncertainty analysis. Probabilistic models may potentially produce more realistic exposure assessment by considering the whole distribution of the model parameters, representing real variation. Meanwhile, the probabilistic model is subjected to uncertainties and a Monte Carlo method is typically used as a computational tool to illustrate approximate uncertainty and variability.

## 5. Conclusions

The occurrence of FQ residues was determined in honey samples from Zhejiang Province in China using PRiME HLB cleanup coupled with LC-MS/MS. Norfloxacin and ciprofloxacin were the major contaminants. Most positive samples contained single FQ residues, whereas the co-occurrence of target compounds was hardly found in the honey. The detection frequency of FQs in plain areas was higher than in mountainous areas. Both detection frequency and residue levels of FQs in honey from *Apis mellifera* were higher than those in indigenous honey from *Apis cerana*. The detection frequency and residue levels of FQs in rape honey were highest among the four most productive honey species in China. Processed honey from commercial sale channels had a higher detection frequency of FQ residues than raw honey from apiaries. 

The deterministic exposure assessment showed that the potential risks of FQ residues in honey from *Apis mellifera* were higher than from *Apis cerana*. The potential risks of FQ residues in Chinese milk vetch honey and honey from supermarkets were relatively high for consumers. The probabilistic exposure assessment indicated that the dietary risk for adults was high among all age groups. Sensitivity analysis showed that FQ concentration was the most sensitive factor for noncarcinogenic risks. Both the current deterministic and the probabilistic exposure assessment indicated that the health risk of FQ residues in honey was low for human health. This study serves as a useful reference for understanding the trouble of antibiotic residues in honey and enriching the research on emerging pollutants in honey products.

## Figures and Tables

**Table 1:** Validation of analytical method for each compound in honey at the spiked level.

Compound	Retention Time (min)	Linearity (μg/L)	R2	LOD (μg/kg)	LOQ (μg/kg)	Matrix Effect (%)
marbofloxacin	7.21	0.1~200	0.994	0.17	0.54	104.6
fleroxacin	7.43	0.1~200	0.998	0.58	1.75	95.6
ofloxacin	7.88	0.1~200	0.999	0.15	0.54	99.4
pefloxacin	7.91	0.1~200	0.999	0.15	0.53	110.1
enoxacin	7.94	0.1~200	0.999	0.21	0.63	106.4
norfloxacin	8.18	0.1~200	0.999	0.35	1.0	103.8
ciprofloxacin	8.38	0.1~200	0.999	0.30	1.0	101.2
enrofloxacin	8.48	0.1~200	0.991	0.47	1.4	103.6
danofloxacin	8.49	0.1~200	0.996	0.28	0.85	92.9
lomefloxacin	8.64	0.1~200	0.999	1.03	3.10	106.4
difloxacin	8.74	0.1~200	0.992	0.16	0.68	96.4
sarafloxacin	8.89	0.1~200	0.998	0.2	0.59	104.7
flumequine	10.57	0.1~200	0.999	0.37	1.05	104.6

**Table 2:** Deterministic assessment of exposure to risky FQs through ingesting honey among consumers.

Compound	ADI (μg/kg)	Average Residue (μg/kg)	EDI (μg/kg bw/Day)	HQ
Ciprofloxacin	0.15 [22]	1.06 ± 10.88	1.77 × 10^−4^	1.18 × 10^−3^
Enrofloxacin	6.2 [20]	0.24 ± 0.091	4.0 × 10^−5^	6.45 × 10^−6^
Norfloxacin	14 [21]	16.10 ± 307.76	2.68 × 10^−3^	1.91 × 10^−4^
Ofloxacin	3.2 [22]	0.091 ± 0.422	1.52 × 10^−5^	4.75 × 10^−6^

**Table 3:** Deterministic assessment of exposure to risky FQs through ingesting Western and Chinese honeybee honey among consumers.

Compound	Entomological Origin	ADI (μg/kg)	Average Residue (μg/kg)	EDI (μg/kg w/Day)	HQ
Ciprofloxacin	*Apis mellifera*	0.15 [22]	1.32 ± 12.4	2.19 × 10^−4^	1.46 × 10^−3^
	*Apis cerana*		0.21 ± 0.51	3.45 × 10^−5^	2.30 × 10^−4^
Enrofloxacin	*Apis mellifera*	6.2 [20]	0.240 ± 0.1	4.0 × 10^−5^	6.45 × 10^−6^
	*Apis cerana*		0.235	3.92 × 10^−3^	6.32 × 10^−6^
Norfloxacin	*Apis mellifera*	14 [21]	20.7 ± 351.7	3.45 × 10^−3^	2.46 × 10^−4^
	*Apis cerana*		1.113 ± 7.5	1.86 × 10^−4^	1.33 × 10^−5^
Ofloxacin	*Apis mellifera*	3.2 [22]	0.101 ± 0.48	1.68 × 10^−5^	5.25 × 10^−6^
	*Apis cerana*		0.075	1.25 × 10^−5^	3.91 × 10^−6^

**Table 4:** Deterministic assessment of exposure to risky FQs through ingesting various floral origins of honey among consumers.

Compound	Floral Origin	ADI (μg/kg)	Average Residue (μg/kg)	EDI (μg/kg bw/Day)	HQ
Ciprofloxacin	Acacia	0.15 [22]	0.833 ± 6.25	1.39 × 10^−5^	9.27 × 10^−5^
	Chinese milk vetch		74.3 ± 92.7	1.24 × 10^−2^	8.27 × 10^−2^
	Loquat		2.12 ± 11.6	3.53 × 10^−4^	3.80 × 10^−3^
	Mutiflower		0.366 ± 1.78	6.10 × 10^−5^	4.07 × 10^−4^
	Rape		0.4 ± 2.64	6.70 × 10^−5^	4.47 × 10^−4^
Enrofloxacin	Rape	6.2 [20]	0.245 ± 0.16	4.10 × 10^−5^	6.61 × 10^−6^
Norfloxacin	Acacia	14 [21]	3.15 ± 2.78	5.25 × 10^−4^	3.75 × 10^−5^
	Chinese milk vetch		0.82 ± 1.29	1.37 × 10^−4^	9.79 × 10^−4^
	Citrus		0.631 ± 1.82	1.05 × 10^−4^	7.50 × 10^−6^
	Honeysuckle Flowers		17.5 ± 15.6	2.92 × 10^−3^	2.09 × 10^−6^
	Loquat		0.573 ± 2.25	9.60 × 10^−5^	6.86 × 10^−6^
	Mutiflower		9.66 ± 104.8	1.61 × 10^−3^	1.15 × 10^−4^
	Rape		34.8 ± 504.1	5.80 × 10^−3^	4.14 × 10^−4^
Ofloxacin	Rape	3.2 [22]	0.13 ± 0.70	2.20 × 10^−5^	6.88 × 10^−6^

**Table 5:** Deterministic assessment of exposure to risky FQs through ingesting honey from different sale channels among consumers.

Compound	Sale Channel	ADI (μg/kg)	Average Residue (μg/kg)	EDI (μg/kg bw/Day)	HQ
Ciprofloxacin	Apiary	0.15 [22]	0.34 ± 2.18	5.63 × 10^−5^	3.75 × 10^−4^
	Supermarket		1.16 ± 7.44	1.93 × 10^−4^	1.29 × 10^−3^
	Honey processing facility		6.65 ± 32.27	1.11 × 10^−4^	7.40 × 10^−4^
	Bee product store		0.21 ± 0.41	3.57 × 10^−5^	2.50 × 10^−4^
Enrofloxacin	Apiary	6.2 [20]	0.24 ± 0.11	4.0 × 10^−5^	6.45 × 10^−6^
Norfloxacin	Apiary	14 [21]	17.4 ± 353.61	2.9 × 10^−3^	2.07 × 10^−4^
	Supermarket		10.3 ± 48.3	1.72 × 10^−3^	1.23 × 10^−4^
	Honey processing facility		0.72 ± 2.64	1.22 × 10^−4^	8.71 × 10^−6^
	Bee product store		35.1 ± 223.93	5.85 × 10^−3^	4.18 × 10^−4^
Ofloxacin	Apiary	3.2 [22]	0.102 ± 0.49	1.70 × 10^−5^	5.30 × 10^−6^

**Table 6:** Probabilistic assessment of exposure to risky FQs through ingesting honey among consumers.

Compound	Percentile	Dietary Exposure Different Age Groups (HQs)			
		Children (4–11 Years)	Adolescents (12–18 Years)	Adults (19–64 Years)	Older Adults over 65 Years
Ciprofloxacin	P50	2.42 × 10^−2^	5.55 × 10^−2^	0.117	0.107
	P95	7.87 × 10^−2^	0.102	0.217	0.199
Enrofloxacin	P50	1.34 × 10^−5^	1.77 × 10^−5^	3.77 × 10^−5^	3.50 × 10^−5^
	P95	2.39 × 10^−5^	3.08 × 10^−5^	5.92 × 10^−5^	6.00 × 10^−5^
Norfloxacin	P50	1.66 × 10^−2^	2.16 × 10^−2^	4.59 × 10^−2^	4.24 × 10^−2^
	P95	2.94 × 10^−2^	4.01 × 10^−2^	8.57 × 10^−2^	7.86 × 10^−2^
Ofloxacin	P50	1.02 × 10^−4^	1.34 × 10^−4^	2.87 × 10^−4^	2.64 × 10^−4^
	P95	1.94 × 10^−4^	2.50 × 10^−4^	5.38 × 10^−4^	4.88 × 10^−4^

## Data Availability

The data presented in this study are available in the article.

## Supplementary Materials

The following supporting information can be downloaded at https://www.mdpi.com/article/10.3390/toxics11090744/s1, Table S1 Floral origin and sale channel of samples collected in Zhejiang Province, China; Table S2: Information regarding the sampling strategy in detail; Table S3: The MRM (Multiple reaction monitoring) transition parameters for FQs; Table S4: Exposure parameters used to perform probabilistic risk assessments of human exposure to FQs in honey; Table S5: The results of FQ residues in honey from different regions Zhejiang Province, China; Table S6: The results of FQ residues in honey from different Years in Zhejiang Province, China.

## References

[1] Chen L.H., Wu J., Zhang F.X. (2014). Analysis of domestic bee products market of 2013. China Anim. Ind..

[2] Reybroeck W., Daeseleire E., De Brabander H.F., Herman L. (2012). Antimicrobials in beekeeping. Vet. Microbiol..

[3] Chiesa L.M., Panseri S., Nobile M., Ceriani F., Arioli F. (2018). Distribution of POPs, pesticides and antibiotic residues in organic honeys from different production areas. Food Addit. Contam. Part. A Chem. Anal. Control Expo. Risk Assess..

[4] Tillotson G.S., Doern G.V., Blondeau J.M. (2006). Optimal antimicrobial therapy: The balance of potency and exposure. Expert. Opin. Investig. Drugs.

[5] Muaz K., Riaz M., Akhtar S., Park S., Ismail A. (2018). Antibiotic Residues in Chicken Meat: Global Prevalence, Threats, and Decontamination Strategies: A Review. J. Food Prot..

[6] Xu W., Zhang G., Li X., Zou S., Li P., Hu Z., Li J. (2007). Occurrence and elimination of antibiotics at four sewage treatment plants in the Pearl River Delta (PRD), South China. Water Res..

[7] Huyghebaert G., Ducatelle R., Van Immerseel F. (2011). An update on alternatives to antimicrobial growth promoters for broilers. Vet. J..

[8] Pereira A., Silva L.J.G., Rodrigues J., Lino C., Pena A. (2018). Risk assessment of fluoroquinolones from poultry muscle consumption: Comparing healthy adult and pre-school populations. Food Chem. Toxicol..

[9] MAPRC (2015). Decision on the prohibition of four veterinary drugs (lomefloxacin, pefloxacin, ofloxacin and norfloxacin) in food animals. Ministry of Agriculture of the People’s Republic of China Bulletin No. 2292, China.

[10] Mottier P., Hammel Y.A., Gremaud E., Guy P.A. (2008). Quantitative high-throughput analysis of 16 (fluoro)quinolones in honey using automated extraction by turbulent flow chromatography coupled to liquid chromatography-tandem mass spectrometry. J. Agric. Food Chem..

[11] Jin Y., Zhang J., Zhao W., Zhang W., Wang L., Zhou J., Li Y. (2017). Development and validation of a multiclass method for the quantification of veterinary drug residues in honey and royal jelly by liquid chromatography-tandem mass spectrometry. Food Chem..

[12] Wang Y.P., Dong X.L., Han M.H., Yang Y., Qian L., Huang M., Luo B.Z., Wang H.X., Chen Y., Jiang Q.W. (2022). Antibiotic residues in honey in the Chinese market and human health risk assessment. J. Hazard. Mater..

[13] Soares S., Grazina L., Mafra I., Costa J., Pinto M.A., Duc H.P., Oliveira M., Amaral J.S. (2008). Novel diagnostic tools for Asian (*Apis cerana*) and European (*Apis mellifera*) honey authentication. Food Res. Int..

[14] Liu N.N., Liu H.M., Yan J., Li X.G., Li Y., Wang T.J., He J.M., Niu Q.S., Xing X.M. (2022). Geometric morphology and population genomics provide insights into the adaptive evolution of *Apis cerana* in Changbai Mountain. BMC Genom..

[15] Xu R., Cheng N., Huang W., Gao H., Deng J.D., Cao W. (2012). Effects of the processing steps on parathion levels during honey. Food Control..

[16] Zheng H.Q., Cao L.F., Huang S.K., Neumann P., Hu F.L. (2018). Current Status of the Beekeeping Industry in China. Asian Beekeeping in the 21st Century.

[17] Wang J.M., Yang H., Zeng Y.H., Wu L.Q., Li R., Yuan Y.W., Qiang M.R. (2018). Measuring the antibiotics and pesticides in honeys by ultra-performance liquid chromatography tandem mass spectrometry (UPLC-MS/MS). J. ZheJIang Univ..

[18] Zheng H.Q., Wei W.T., Hu F.L. (2015). Beekeeping Industry In China. Bee World.

[19] Ji X.F., Xu Y., Wang J.M., Lyu W.T., Li R., Tan S., Xiao Y.P., Tang B., Yang H., Qian M.R. (2021). Multiresidue determination of antibiotics in ready-to-eat duck eggs marketed through e-commerce stores in China and subsequent assessment of dietary risks to consumers. J. Food Sci..

[20] (2019). National Food Safety Standard-Maximum Residue Limits for Veterinary Drugs in Foods.

[21] (2022). National Food Safety Standard Maximum Residue Limits of 41 Veterinary Drugs in Food.

[22] Wang H.X., Wang N., Qian J.H., Hu L.Y., Huang P.X., Su M.F., Yu X., Fu C.W., Jiang F., Zhao Q. (2017). Urinary antibiotics of pregnant women in Eastern China and cumulative health risk assessment. Environ. Sci. Technol..

[23] FAO/WHO (2017). Report of the JECFA/JMPR Expert Working Group on Harmonizing/Combining Exposure from Veterinary Drug and Pesticide Use.

[24] GEMS/FOOD (2013). Global Environment Monitoring System—Food Contamination Monitoring and Assessment Programme, GEMS/Food Consumption Cluster Diets.

[25] Wang X., Goulson D., Chen L.Z., Zhang J.Z., Zhao W., Jin Y., Yang S.P., Li Y., Zhou J.H. (2020). Occurrence of Neonicotinoids in Chinese Apiculture and a Corresponding Risk Exposure Assessment. Environ. Sci. Technol..

[26] Jiang C.L., Zhao Q., Zheng L.G., Chen X., Li C., Ren M.X. (2021). Distribution, source and health risk assessment based on the Monte Carlo method of heavy metals in shallow groundwater in an area affected by mining activities China. Ecotoxicol. Environ. Saf..

[27] Alipour M., Sarafraz M., Chavoshi H., Bay A., Nematollahi A., Sadani M., Fakhri Y., Vasseghian Y., Khaneghah A.M. (2020). The concentration and probabilistic risk assessment of potentially toxic elements in fillets of silver pomfret (*Pampus argenteus*): A global systematic review and meta-analysis. J. Environ. Sci..

[28] Soltanpour Z., Mohammadian Y., Fakhri Y. (2021). The concentration of benzene, toluene, ethylbenzene, and xylene in ambient air of the gas stations in Iran: A systematic review and probabilistic health risk assessment. Toxicol. Ind. Health.

[29] Kujawski M.W., Namiesnik J. (2008). Challenges in preparing honey samples for chromatographic determination of contaminants and trace residues. TrAC Trends Anal. Chem..

[30] Fukumitsu T., Waki M., Hagio M., Hayashi T., Kuwahara C. (2021). Development of an Analytical Method for Simultaneous Determinationof Quinolones and Tetracyclines in Livestock and Fishery Products. Shokuhin Eiseigaku Zasshi.

[31] Roberta G., Giorgio S., Danilo G., Rosanna R., Simone M. (2015). Multiclass determination of 27 antibiotics in honey. Food Control..

[32] Louppis A.P., Kontominas M.G., Papastephanou C. (2017). Determination of antibiotic residues in honey by high-performance liquid chromatography with electronspray ionization tandem mass spectrometry. Food Anal. Methods.

[33] Reybroeck W. (2018). Residues of antibiotics and chemotherapeutics in honey. J. Apic. Res..

[34] Yatsukawa Y., Ito H., Matsuda T., Nakamura M., Watai M., Fujita K. (2011). Determination of residual fluoroquinolones in honey by liquid chromatography using metal chelate affinity chromatography. J. AOAC Int..

[35] Bonerba E., Panseri S., Arioli F., Nobile M., Terio V., Di Cesare F., Tantillo G., Maria Chiesa L. (2020). Determination of antibiotic residues in honey in relation to different potential sources and relevance for food inspection. Food Chem..

[36] Shao Y., Wang Y., Yuan Y., Xie Y. (2021). A systematic review on antibiotics misuse in livestock and aquaculture and regulation implications in China. Sci. Total Environ..

[37] Lei H.Y., Guo J.B., Lv Z., Zhu X.H., Xue X.M., Wu L., Cao W. (2018). Simultaneous Determination of Nitroimidazoles and Quinolones in Honey by Modified QuEChERS and LC-MS/MS Analysis. Int. J. Anal. Chem..

[38] Mahmoudi R., Norian R., Pajohi-Alamoti M. (2014). Antibiotic residues in Iranian honey by ELISA. Int. J. Food Prop..

[39] Er Demirhan B., Demirhan B. (2022). Detection of Antibiotic Residues in Blossom Honeys from Different Regions in Turkey by LC-MS/MS Method. Antibiotics.

[40] Xue Y. (2007). Study on Thermostability of Chinese Monofloral Honey. Master’s Thesis.

[41] Vanengelsdorp D., Meixner M.D. (2010). A historical review of managed honey bee populations in Europe and the United States and the factors that may affect them. J. Invertebr. Pathol..

[42] Yuan Y., Deng Z., Zhang B., Li G., Zhang J., Liu R., Li H. (2021). Quality evaluation and geographical classification of immature rape and acacia honeys in China. J. Sci. Food Agric..

[43] Yuan Y.W., Chen C., Zheng C.M., Wang X.L., Yang G.L., Wang Q., Zhang Z.H. (2014). Residue of chlorpyrifos and cypermethrin in vegetables and probabilistic exposure assessment for consumers in Zhejiang Province, China. Food Control..

[44] Mandell L., Tillotson G. (2002). Safety of fluoroquinolones: An update. Can. J. Infect. Dis..

[45] Kim D., Song N., Nam T.G., Jung Y.S., Yoo M. (2021). Investigation and human health risk assessment of multi-class veterinary antibiotics in honey from South Korea. J. Food Compost. Anal..

[46] Bwatanglang I.B., Bimba J.S., Magili S., Musa Y., Zira S. (2019). Dietary exposure to antibiotics residue in honey and the potential health risks to consumers in Adamawa state, Nigeria. Int. J. Chem. Stud..

[47] Stephenson C.L., Harris C.A. (2016). An assessment of dietary exposure to glyphosate using refined deterministic and probabilistic methods. Food Chem. Toxicol..

[48] Chu Y.L., Chimeddulam D., Sheen L.Y., Wu K.Y. (2013). Probabilistic risk assessment of exposure to leucomalachite green residues from fish products. Food Chem. Toxicol..

[49] Kennedy M.C., Van der Voet H., Roelofs V.J., Roelofs W., Glass C.R., de Boer W.J., Kruisselbrink J.W., Hart A.D.M. (2015). New approaches to uncertainty analysis for use in aggregate and cumulative risk assessment of pesticides. Food Chem. Toxicol..

[50] Kettler S., Kennedy M., McNamara C., Oberd€orfer R., O’Mahony C., Schnabel J., Smith B., Sprong C., Faludi R., Tennant D. (2015). Assessing and reporting uncertainties in dietary exposure analysis: Mapping of uncertainties in a tiered approach. Food Chem. Toxicol..

[51] Stanton I.C., Murray A.K., Zhang L., Snape J., Gaze W.H. (2020). Evolution of antibiotic resistance at low antibiotic concentrations including selection below the minimal selective concentration. Commun. Biol..

