# Supplementary material for: Comprehensive Investigation of Fluoroquinolone Residues in Apis mellifera and Apis cerana Honey and Potential Risks to Consumers: A Five-Year Study (2014–2018) in Zhejiang Province, China
Source: Toxics. 2023 Aug 31;11(9):744. doi: 10.3390/toxics11090744 (PMC10536307; doi:10.3390/toxics11090744)
Supplement: Supplementary file 1 [file toxics-11-00744-s001.zip › toxics-2553963-supplementary.pdf]

Comprehensive investigation of fluoroquinolone residues in *Apis mellifera* and *Apis cerana* honey and potential risks to consumers: A five-year study (2014–2018) in Zhejiang Province, China

Liang He [1], Leiding Shen [2], Jie Zhang [3] and Rui Li [4,*]

[1] Animal Experiment Center; Institute of Animal Husbandry and Veterinary Science, Zhejiang Academy of Agricultural Sciences, Hangzhou 310021, China; xueqiguorong@163.com

[2] Agricultural Economic Service Center, Shimen town, Tongxiang city, Jiaxing 314512, Zhejiang, China; shenleidingtxsm@163.com

[3] Tongxiang Institute of Agricultural Sciences, Jiaxing Academy of Agricultural Sciences, Jiaxing 314512, Zhejiang, China; zj1818zi@163.com

[4] Institute of Agro-product Safety and Nutrition, Zhejiang Academy of Agricultural Sciences；Agricultural Ministry Key Laboratory for Pesticide Residue Detection, Hangzhou 310021, China; microvet@163.com

* Correspondence: microvet@163.com

Table (S1–S6) Contents

**Table S1**	**Floral origin and sale channel of samples collected in Zhejiang Province, China**
**Table S2**	**Information regarding the sampling strategy in detail;**
**Table S3**	**The MRM (Multiple reaction monitoring) transition parameters for FQs;**
**Table S4**	**Exposure parameters used to perform probabilistic risk assessments of human exposure to FQs in honey.**
**Table S5**	**The results of FQ residues in honey from different regions Zhejiang Province, China.**
**Table S6**	**The results of FQ residues in honey from different Years in Zhejiang Province, China.**

Supporting information

Table S1. Floral origin and sale channel of samples collected in Zhejiang Province, China.

	Sale channel				
**Floral origin**	**Apiary**	**Supermarket**	**Honey processing facility**	**Bee-product store**	**Total**
Acacia (Robinia. pseudoacacia L.*)*	21	40	25	18	104
*Chaste (*Vitex. negundo L.*)*	3	0	4	0	7
*Cherry (*Prunus pseudocerasus (Lindl*).)*	1	0	0	0	1
Chestnut (Castanea mollissima BL.)	2	0	0	0	2
China Soapberry (*Sapindus saponaria Linnaeus*)	8	0	1	0	9
Chinese Bellflower（*Platycodon grandiflorus (Jacq.) A.DC*)	0	0	1	0	1
Chinese Magnoliavine Fruit *(*Schisandra) Michx.chinensis (Turcz.Baill*)*	1	0	0	0	1
Chinese milk vetch (*Astragalus sinicus L.*)	0	2	3	0	5
Chrysanthemum Ramat Tzvel (*Dendranthema morifolium)*	1	2	0	0	3

Citrus (*Citrus reticulata Blanco*)	14	1	1	1	17
Common Eurya (*Eurya japonica Thunb*)	1	0	0	0	1
Herb of Dahurian Loosestrife (*Lysimachia davurica Ledebour*)	0	0	1	0	1
Honeysuckle Flowers (*Lonicera japonica Thunb.*）	0	2	0	0	2
Jujube (*Ziziphus jujuba Mill*)	4	2	2	2	10
Linden (*Tilia. amurensis Rupr*)	8	4	0	2	14
Longan *(Dimocarpus longan Lour*)	0	1	0	0	1
Loquat (*Eriobotrya japonica (Thunb.) Lindl)*	19	6	6	2	33
Lucerne (*Medicago sativa L.*)	1	0	0	0	1
Lychee (*Litchi chinensis Sonn.*)	0	2	0	0	2
Medlar *(Lycium barbarum L.*)	0	0	1	0	1
Motherwort (*Leonurus japonicus Houtt.*)	0	1	1	1	3
Mutiflower	186	6	9	11	212
Quillaja saponaria	1	0	0	0	1

Rape (*Brassica. campestris L*)	228	0	12	4	244
Sunflower (*Helianthus annuus L.*)	1	0	0	0	1
Sweet Osmanthus (*Osmanthus fragrans*)	2	1	0	1	4

Table S2 Information regarding the sampling strategy in detail.

Samples	Entomological origins	Years	Floral origins	Sale channels	Regions	Geographical coordinates
Honey-001	*Apis mellifera*	2014	Acacia	processing facility	Quzhou	118° 63'E, 28° 74'N
Honey-002	*Apis mellifera*	2014	Acacia	processing facility	Quzhou	118° 63'E, 28° 74'N
Honey-003	*Apis mellifera*	2014	Acacia	processing facility	Quzhou	118° 63'E, 28° 74'N
Honey-004	*Apis mellifera*	2014	Acacia	processing facility	Quzhou	118° 63'E, 28° 74'N
Honey-005	*Apis mellifera*	2014	Mutiflower	apiary	Quzhou	118° 63'E, 28° 74'N
Honey-006	*Apis mellifera*	2014	Chinese milk vetch	processing facility	Quzhou	118° 63'E, 28° 74'N
Honey-007	*Apis mellifera*	2014	Acacia	processing facility	Quzhou	118° 63'E, 28° 74'N
Honey-008	*Apis mellifera*	2014	Medlar	processing facility	Quzhou	118° 63'E, 28° 74'N
Honey-009	*Apis mellifera*	2014	Jujube	processing facility	Quzhou	118° 63'E, 28° 74'N
Honey-010	*Apis mellifera*	2014	Longan	processing facility	Quzhou	118° 63'E, 28° 74'N
Honey-011	*Apis cerana*	2014	Mutiflower	processing facility	Quzhou	118° 63'E, 28° 74'N
Honey-012	*Apis mellifera*	2014	Herb of Dahurian	processing facility	Quzhou	118° 63'E, 28° 74'N
Honey-013	*Apis mellifera*	2014	Rape	processing facility	Quzhou	118° 63'E, 28° 74'N
Honey-014	*Apis cerana*	2014	Mutiflower	processing facility	Quzhou	118° 63'E, 28° 74'N
Honey-015	*Apis mellifera*	2014	Rape	processing facility	Quzhou	118° 63'E, 28° 74'N
Honey-016	*Apis mellifera*	2014	Chinese Bellflower	processing facility	Quzhou	118° 63'E, 28° 74'N
Honey-017	*Apis mellifera*	2014	Longan	processing facility	Quzhou	118° 63'E, 28° 74'N
Honey-018	*Apis mellifera*	2014	Chaste	processing facility	Quzhou	118° 63'E, 28° 74'N

Honey-019	*Apis mellifera*	2014	Acacia	processing facility	Quzhou	118° 63'E, 28° 74'N
Honey-020	*Apis mellifera*	2014	Rape	apiary	Quzhou	118° 63'E, 28° 74'N
Honey-021	*Apis mellifera*	2014	Chrysanthemum	apiary	Quzhou	118° 63'E, 28° 74'N
Honey-022	*Apis mellifera*	2014	Mutiflower	apiary	Quzhou	118° 63'E, 28° 74'N
Honey-023	*Apis mellifera*	2014	Mutiflower	bee-product stores	Quzhou	118° 63'E, 28° 74'N
Honey-024	*Apis mellifera*	2014	Acacia	bee-product stores	Quzhou	118° 63'E, 28° 74'N
Honey-025	*Apis cerana*	2014	Mutiflower	bee-product stores	Quzhou	118° 63'E, 28° 74'N
Honey-026	*Apis mellifera*	2014	Acacia	bee-product stores	Quzhou	118° 63'E, 28° 74'N
Honey-027	*Apis mellifera*	2014	Mutiflower	bee-product stores	Quzhou	118° 63'E, 28° 74'N
Honey-028	*Apis mellifera*	2014	Longan	bee-product stores	Quzhou	118° 63'E, 28° 74'N
Honey-029	*Apis mellifera*	2014	Longan	supermarket	Quzhou	118° 63'E, 28° 74'N
Honey-030	*Apis mellifera*	2014	Motherwort	supermarket	Quzhou	118° 63'E, 28° 74'N
Honey-031	*Apis mellifera*	2014	HoneySuchle Flowers	supermarket	Quzhou	118° 63'E, 28° 74'N
Honey-032	*Apis mellifera*	2014	Lychee	supermarket	Quzhou	118° 63'E, 28° 74'N
Honey-033	*Apis mellifera*	2014	Acacia	supermarket	Quzhou	118° 63'E, 28° 74'N
Honey-034	*Apis mellifera*	2014	Longan	supermarket	Quzhou	118° 63'E, 28° 74'N
Honey-035	*Apis mellifera*	2014	Linden	apiary	Lishui	120° 09'E, 28° 66'N
Honey-036	*Apis mellifera*	2014	Acacia	apiary	Lishui	120° 09'E, 28° 66'N
Honey-037	*Apis mellifera*	2014	Chaste	apiary	Lishui	120° 09'E, 28° 66'N
Honey-038	*Apis mellifera*	2014	Longan	apiary	Lishui	120° 09'E, 28° 66'N
Honey-039	*Apis mellifera*	2014	HoneySuchle Flowers	supermarket	Lishui	120° 09'E, 28° 66'N
Honey-040	*Apis mellifera*	2014	Chrysanthemum	supermarket	Lishui	120° 09'E, 28° 66'N

Honey-041	*Apis mellifera*	2014	Acacia	supermarket	Lishui	120° 09'E, 28° 66'N
Honey-042	*Apis mellifera*	2014	Mutiflower	supermarket	Lishui	120° 09'E, 28° 66'N
Honey-043	*Apis mellifera*	2014	Mutiflower	supermarket	Lishui	120° 09'E, 28° 66'N
Honey-044	*Apis mellifera*	2014	Citrus	supermarket	Lishui	120° 09'E, 28° 66'N
Honey-045	*Apis cerana*	2014	Longan	apiary	Lishui	120° 09'E, 28° 66'N
Honey-046	*Apis mellifera*	2014	Mutiflower	supermarket	Lishui	120° 09'E, 28° 66'N
Honey-047	*Apis mellifera*	2014	Acacia	bee-product stores	Ningbo	121° 27'E, 30° 17'N
Honey-048	*Apis mellifera*	2014	Linden	supermarket	Ningbo	121° 27'E, 30° 17'N
Honey-049	*Apis mellifera*	2014	Acacia	supermarket	Ningbo	121° 27'E, 30° 17'N
Honey-050	*Apis mellifera*	2014	Longan	supermarket	Ningbo	121° 27'E, 30° 17'N
Honey-051	*Apis mellifera*	2014	Chinese milk vetch	supermarket	Ningbo	121° 27'E, 30° 17'N
Honey-052	*Apis mellifera*	2014	Linden	apiary	Jinhua	119° 46'E, 29° 21'N
Honey-053	*Apis mellifera*	2014	Acacia	apiary	Jinhua	119° 46'E, 29° 21'N
Honey-054	*Apis mellifera*	2014	Chaste	apiary	Jinhua	119° 46'E, 29° 21'N
Honey-055	*Apis mellifera*	2014	Acacia	apiary	Jinhua	119° 46'E, 29° 21'N
Honey-056	*Apis mellifera*	2014	Longan	supermarket	Jinhua	119° 46'E, 29° 21'N
Honey-057	*Apis mellifera*	2014	Linden	supermarket	Jinhua	119° 46'E, 29° 21'N
Honey-058	*Apis mellifera*	2014	Chrysanthemum	supermarket	Jinhua	119° 46'E, 29° 21'N
Honey-059	*Apis mellifera*	2014	Longan	bee-product stores	Jinhua	119° 46'E, 29° 21'N
Honey-060	*Apis cerana*	2014	Mutiflower	apiary	Hangzhou	119° 96'E, 30° 05'N
Honey-061	*Apis mellifera*	2014	Rape	apiary	Hangzhou	119° 96'E, 30° 05'N
Honey-062	*Apis mellifera*	2014	Rape	apiary	Hangzhou	119° 96'E, 30° 05'N

Honey-063	*Apis mellifera*	2014	Rape	apiary	Hangzhou	119° 96'E, 30° 05'N
Honey-064	*Apis mellifera*	2014	Acacia	supermarket	Hangzhou	119° 96'E, 30° 05'N
Honey-065	*Apis mellifera*	2014	Acacia	supermarket	Hangzhou	119° 96'E, 30° 05'N
Honey-066	*Apis mellifera*	2014	Acacia	supermarket	Hangzhou	119° 96'E, 30° 05'N
Honey-067	*Apis mellifera*	2014	Acacia	supermarket	Hangzhou	119° 96'E, 30° 05'N
Honey-068	*Apis mellifera*	2014	Linden	supermarket	Hangzhou	119° 96'E, 30° 05'N
Honey-069	*Apis mellifera*	2014	Acacia	supermarket	Hangzhou	119° 96'E, 30° 05'N
Honey-070	*Apis mellifera*	2014	Linden	apiary	Hangzhou	119° 69'E, 29° 80'N
Honey-071	*Apis mellifera*	2014	Acacia	apiary	Hangzhou	119° 69'E, 29° 80'N
Honey-072	*Apis mellifera*	2014	Mutiflower	apiary	Hangzhou	119° 69'E, 29° 80'N
Honey-073	*Apis mellifera*	2014	Sunflower	apiary	Hangzhou	119° 69'E, 29° 80'N
Honey-074	*Apis mellifera*	2014	Acacia	bee-product stores	Hangzhou	119° 69'E, 29° 80'N
Honey-075	*Apis mellifera*	2014	Rape	apiary	Hangzhou	119° 69'E, 29° 80'N
Honey-076	*Apis mellifera*	2014	Rape	apiary	Hangzhou	119° 69'E, 29° 80'N
Honey-077	*Apis mellifera*	2014	Jujube	supermarket	Hangzhou	119° 69'E, 29° 80'N
Honey-078	*Apis mellifera*	2014	Acacia	supermarket	Hangzhou	119° 69'E, 29° 80'N
Honey-079	*Apis mellifera*	2014	Acacia	supermarket	Hangzhou	119° 69'E, 29° 80'N
Honey-080	*Apis mellifera*	2014	Chinese milk vetch	supermarket	Hangzhou	119° 69'E, 29° 80'N
Honey-081	*Apis mellifera*	2014	Acacia	supermarket	Hangzhou	119° 69'E, 29° 80'N
Honey-082	*Apis cerana*	2014	Mutiflower	supermarket	Hangzhou	119° 69'E, 29° 80'N
Honey-083	*Apis mellifera*	2015	Acacia	processing facility	Quzhou	118° 63'E, 28° 74'N
Honey-084	*Apis mellifera*	2015	Rape	processing facility	Quzhou	118° 63'E, 28° 74'N

Honey-085	*Apis mellifera*	2015	Citrus	processing facility	Quzhou	118° 63'E, 28° 74'N
Honey-086	*Apis mellifera*	2015	Longan	processing facility	Quzhou	118° 63'E, 28° 74'N
Honey-087	*Apis mellifera*	2015	Acacia	processing facility	Quzhou	118° 63'E, 28° 74'N
Honey-088	*Apis mellifera*	2015	Rape	apiary	Quzhou	118° 63'E, 28° 74'N
Honey-089	*Apis mellifera*	2015	Rape	apiary	Quzhou	118° 63'E, 28° 74'N
Honey-090	*Apis mellifera*	2015	Rape	apiary	Quzhou	118° 63'E, 28° 74'N
Honey-091	*Apis mellifera*	2015	Rape	apiary	Quzhou	118° 63'E, 28° 74'N
Honey-092	*Apis mellifera*	2015	Rape	apiary	Quzhou	118° 63'E, 28° 74'N
Honey-093	*Apis mellifera*	2015	Rape	apiary	Quzhou	118° 63'E, 28° 74'N
Honey-094	*Apis mellifera*	2015	Rape	apiary	Quzhou	118° 63'E, 28° 74'N
Honey-095	*Apis mellifera*	2015	Rape	apiary	Quzhou	118° 63'E, 28° 74'N
Honey-096	*Apis mellifera*	2015	Rape	apiary	Quzhou	118° 63'E, 28° 74'N
Honey-097	*Apis mellifera*	2015	Rape	apiary	Quzhou	118° 63'E, 28° 74'N
Honey-098	*Apis mellifera*	2015	Rape	apiary	Quzhou	118° 63'E, 28° 74'N
Honey-099	*Apis mellifera*	2015	Rape	apiary	Quzhou	118° 63'E, 28° 74'N
Honey-100	*Apis mellifera*	2015	Rape	apiary	Quzhou	118° 51'E, 28° 90'N
Honey-101	*Apis mellifera*	2015	Rape	apiary	Quzhou	118° 63'E, 28° 74'N
Honey-102	*Apis mellifera*	2015	Rape	apiary	Quzhou	118° 51'E, 28° 90'N
Honey-103	*Apis mellifera*	2015	Rape	apiary	Quzhou	118° 63'E, 28° 74'N
Honey-104	*Apis mellifera*	2015	Rape	apiary	Quzhou	118° 63'E, 28° 74'N
Honey-105	*Apis mellifera*	2015	Rape	apiary	Quzhou	118° 63'E, 28° 74'N
Honey-106	*Apis mellifera*	2015	Rape	apiary	Quzhou	118° 63'E, 28° 74'N

Honey-107	*Apis mellifera*	2015	Rape	apiary	Quzhou	118° 63'E, 28° 74'N
Honey-108	*Apis mellifera*	2015	Acacia	processing facility	Hangzhou	119° 69'E, 29° 80'N
Honey-109	*Apis mellifera*	2015	Rape	processing facility	Hangzhou	119° 69'E, 29° 80'N
Honey-110	*Apis mellifera*	2015	Acacia	processing facility	Hangzhou	119° 69'E, 29° 80'N
Honey-111	*Apis mellifera*	2015	Acacia	processing facility	Hangzhou	119° 69'E, 29° 80'N
Honey-112	*Apis mellifera*	2015	Acacia	apiary	Hangzhou	119° 69'E, 29° 80'N
Honey-113	*Apis mellifera*	2015	Rape	apiary	Hangzhou	119° 69'E, 29° 80'N
Honey-114	*Apis mellifera*	2015	Rape	apiary	Hangzhou	119° 69'E, 29° 80'N
Honey-115	*Apis mellifera*	2015	Rape	apiary	Hangzhou	119° 69'E, 29° 80'N
Honey-116	*Apis mellifera*	2015	Rape	apiary	Hangzhou	119° 69'E, 29° 80'N
Honey-117	*Apis mellifera*	2015	Rape	apiary	Hangzhou	119° 69'E, 29° 80'N
Honey-118	*Apis mellifera*	2015	Rape	apiary	Hangzhou	119° 69'E, 29° 80'N
Honey-119	*Apis mellifera*	2015	Rape	apiary	Hangzhou	119° 69'E, 29° 80'N
Honey-120	*Apis mellifera*	2015	Rape	apiary	Hangzhou	119° 69'E, 29° 80'N
Honey-121	*Apis mellifera*	2015	Rape	apiary	Hangzhou	119° 69'E, 29° 80'N
Honey-122	*Apis mellifera*	2015	Rape	apiary	Hangzhou	119° 69'E, 29° 80'N
Honey-123	*Apis mellifera*	2015	Rape	apiary	Hangzhou	119° 69'E, 29° 80'N
Honey-124	*Apis mellifera*	2015	Rape	apiary	Hangzhou	119° 69'E, 29° 80'N
Honey-125	*Apis mellifera*	2015	Rape	apiary	Hangzhou	119° 69'E, 29° 80'N
Honey-126	*Apis mellifera*	2015	Rape	apiary	Hangzhou	119° 69'E, 29° 80'N
Honey-127	*Apis mellifera*	2015	Rape	apiary	Hangzhou	119° 69'E, 29° 80'N
Honey-128	*Apis mellifera*	2015	Rape	apiary	Hangzhou	119° 69'E, 29° 80'N

Honey-129	*Apis mellifera*	2015	Rape	apiary	Hangzhou	119° 69'E, 29° 80'N
Honey-130	*Apis mellifera*	2015	Rape	apiary	Hangzhou	119° 69'E, 29° 80'N
Honey-131	*Apis mellifera*	2015	Rape	apiary	Hangzhou	119° 69'E, 29° 80'N
Honey-132	*Apis mellifera*	2015	Rape	apiary	Hangzhou	119° 69'E, 29° 80'N
Honey-133	*Apis mellifera*	2015	Rape	apiary	Shaoxing	120° 48'E, 30° 08'N
Honey-134	*Apis mellifera*	2015	Rape	apiary	Shaoxing	120° 58'E, 30° 00'N
Honey-135	*Apis mellifera*	2015	Rape	apiary	Jiaxing	121° 02'E, 30° 70'N
Honey-136	*Apis mellifera*	2015	Rape	apiary	Jiaxing	121° 02'E, 30° 70'N
Honey-137	*Apis mellifera*	2015	Rape	apiary	Jiaxing	121° 02'E, 30° 70'N
Honey-138	*Apis mellifera*	2015	Rape	apiary	Jiaxing	121° 02'E, 30° 70'N
Honey-139	*Apis mellifera*	2015	Rape	apiary	Jiaxing	121° 02'E, 30° 70'N
Honey-140	*Apis mellifera*	2015	Rape	apiary	Jiaxing	121° 02'E, 30° 70'N
Honey-141	*Apis mellifera*	2015	Rape	apiary	Jiaxing	121° 02'E, 30° 70'N
Honey-142	*Apis mellifera*	2015	Rape	apiary	Jiaxing	121° 02'E, 30° 70'N
Honey-143	*Apis mellifera*	2015	Rape	apiary	Jiaxing	121° 02'E, 30° 70'N
Honey-144	*Apis mellifera*	2015	Rape	apiary	Jiaxing	121° 02'E, 30° 70'N
Honey-145	*Apis mellifera*	2015	Rape	apiary	Jiaxing	121° 02'E, 30° 70'N
Honey-146	*Apis mellifera*	2015	Rape	apiary	Jiaxing	121° 02'E, 30° 70'N
Honey-147	*Apis mellifera*	2015	Rape	apiary	Jiaxing	121° 02'E, 30° 70'N
Honey-148	*Apis mellifera*	2015	Rape	apiary	Jiaxing	121° 02'E, 30° 70'N
Honey-149	*Apis mellifera*	2015	Rape	apiary	Jiaxing	121° 02'E, 30° 70'N
Honey-150	*Apis mellifera*	2015	Rape	apiary	Jiaxing	121° 02'E, 30° 70'N

Honey-151	*Apis mellifera*	2015	Rape	apiary	Jiaxing	121° 02'E, 30° 70'N
Honey-152	*Apis mellifera*	2015	Rape	apiary	Jiaxing	121° 02'E, 30° 70'N
Honey-153	*Apis mellifera*	2015	Rape	apiary	Jiaxing	121° 02'E, 30° 70'N
Honey-154	*Apis mellifera*	2015	Rape	apiary	Jiaxing	121° 02'E, 30° 70'N
Honey-155	*Apis mellifera*	2015	Rape	apiary	Quzhou	118° 42'E, 29° 14'N
Honey-156	*Apis mellifera*	2015	Rape	apiary	Quzhou	118° 42'E, 29° 14'N
Honey-157	*Apis mellifera*	2015	Rape	apiary	Quzhou	118° 42'E, 29° 14'N
Honey-158	*Apis mellifera*	2015	Rape	apiary	Quzhou	118° 42'E, 29° 14'N
Honey-159	*Apis mellifera*	2015	Rape	apiary	Quzhou	118° 42'E, 29° 14'N
Honey-160	*Apis mellifera*	2015	Rape	apiary	Quzhou	118° 42'E, 29° 14'N
Honey-161	*Apis mellifera*	2015	Rape	apiary	Quzhou	118° 42'E, 29° 14'N
Honey-162	*Apis mellifera*	2015	Rape	apiary	Quzhou	118° 42'E, 29° 14'N
Honey-163	*Apis mellifera*	2015	Rape	apiary	Quzhou	118° 42'E, 29° 14'N
Honey-164	*Apis mellifera*	2015	Rape	apiary	Quzhou	118° 42'E, 29° 14'N
Honey-165	*Apis mellifera*	2015	Rape	apiary	Quzhou	118° 42'E, 29° 14'N
Honey-166	*Apis mellifera*	2015	Rape	apiary	Quzhou	118° 42'E, 29° 14'N
Honey-167	*Apis mellifera*	2015	Rape	apiary	Quzhou	118° 42'E, 29° 14'N
Honey-168	*Apis mellifera*	2015	Rape	apiary	Quzhou	118° 42'E, 29° 14'N
Honey-169	*Apis mellifera*	2015	Rape	apiary	Quzhou	118° 42'E, 29° 14'N
Honey-170	*Apis mellifera*	2015	Rape	apiary	Quzhou	118° 42'E, 29° 14'N
Honey-171	*Apis mellifera*	2015	Rape	apiary	Quzhou	118° 42'E, 29° 14'N
Honey-172	*Apis mellifera*	2015	Rape	apiary	Quzhou	118° 42'E, 29° 14'N

Honey-173	*Apis mellifera*	2015	Rape	apiary	Quzhou	118° 42'E, 29° 14'N
Honey-174	*Apis mellifera*	2015	Rape	apiary	Quzhou	118° 42'E, 29° 14'N
Honey-175	*Apis mellifera*	2015	Rape	apiary	Quzhou	118° 42'E, 29° 14'N
Honey-176	*Apis mellifera*	2015	Rape	apiary	Quzhou	118° 42'E, 29° 14'N
Honey-177	*Apis mellifera*	2015	Rape	apiary	Quzhou	118° 42'E, 29° 14'N
Honey-178	*Apis mellifera*	2015	Rape	apiary	Quzhou	118° 42'E, 29° 14'N
Honey-179	*Apis mellifera*	2015	Rape	apiary	Quzhou	118° 42'E, 29° 14'N
Honey-180	*Apis mellifera*	2015	Rape	apiary	Quzhou	118° 42'E, 29° 14'N
Honey-181	*Apis mellifera*	2015	Rape	apiary	Quzhou	118° 42'E, 29° 14'N
Honey-182	*Apis mellifera*	2015	Rape	apiary	Quzhou	118° 42'E, 29° 14'N
Honey-183	*Apis mellifera*	2015	Acacia	processing facility	Ningbo	121° 27'E, 30° 17'N
Honey-184	*Apis mellifera*	2015	Rape	processing facility	Ningbo	121° 27'E, 30° 17'N
Honey-185	*Apis mellifera*	2015	Rape	processing facility	Ningbo	121° 27'E, 30° 17'N
Honey-186	*Apis mellifera*	2015	Rape	processing facility	Ningbo	121° 27'E, 30° 17'N
Honey-187	*Apis mellifera*	2015	Acacia	processing facility	Ningbo	121° 27'E, 30° 17'N
Honey-188	*Apis mellifera*	2015	Rape	apiary	Ningbo	121° 27'E, 30° 17'N
Honey-189	*Apis mellifera*	2015	Rape	apiary	Ningbo	121° 27'E, 30° 17'N
Honey-190	*Apis mellifera*	2015	Rape	apiary	Ningbo	121° 27'E, 30° 17'N
Honey-191	*Apis mellifera*	2015	Rape	apiary	Ningbo	121° 27'E, 30° 17'N
Honey-192	*Apis mellifera*	2015	Rape	apiary	Ningbo	121° 27'E, 30° 17'N
Honey-193	*Apis mellifera*	2015	Rape	apiary	Ningbo	121° 27'E, 30° 17'N
Honey-194	*Apis mellifera*	2015	Rape	apiary	Ningbo	121° 27'E, 30° 17'N

Honey-195	*Apis mellifera*	2015	Rape	apiary	Ningbo	121° 27'E, 30° 17'N
Honey-196	*Apis mellifera*	2015	Rape	apiary	Ningbo	121° 27'E, 30° 17'N
Honey-197	*Apis mellifera*	2015	Rape	apiary	Ningbo	121° 27'E, 30° 17'N
Honey-198	*Apis mellifera*	2015	China Soapberry	apiary	Jinhua	119° 57'E, 29° 10'N
Honey-199	*Apis mellifera*	2015	China Soapberry	apiary	Jinhua	119° 57'E, 29° 10'N
Honey-200	*Apis mellifera*	2015	China Soapberry	apiary	Jinhua	119° 57'E, 29° 10'N
Honey-201	*Apis mellifera*	2015	China Soapberry	apiary	Jinhua	119° 57'E, 29° 10'N
Honey-202	*Apis mellifera*	2015	China Soapberry	apiary	Jinhua	119° 57'E, 29° 10'N
Honey-203	*Apis mellifera*	2015	Rape	apiary	Jinhua	119° 57'E, 29° 10'N
Honey-204	*Apis mellifera*	2015	Citrus	apiary	Jinhua	119° 57'E, 29° 10'N
Honey-205	*Apis mellifera*	2015	Jujube	apiary	Jinhua	119° 57'E, 29° 10'N
Honey-206	*Apis mellifera*	2015	Acacia	processing facility	Jinhua	119° 57'E, 29° 10'N
Honey-207	*Apis mellifera*	2015	Longan	processing facility	Jinhua	119° 57'E, 29° 10'N
Honey-208	*Apis mellifera*	2015	Jujube	processing facility	Jinhua	119° 57'E, 29° 10'N
Honey-209	*Apis mellifera*	2015	Rape	apiary	Jinhua	119° 57'E, 29° 10'N
Honey-210	*Apis mellifera*	2015	Citrus	apiary	Jinhua	119° 57'E, 29° 10'N
Honey-211	*Apis mellifera*	2015	China Soapberry	apiary	Jinhua	119° 46'E, 29° 21'N
Honey-212	*Apis mellifera*	2015	Citrus	apiary	Jinhua	119° 46'E, 29° 21'N
Honey-213	*Apis mellifera*	2015	China Soapberry	apiary	Jinhua	119° 46'E, 29° 21'N
Honey-214	*Apis mellifera*	2015	Rape	processing facility	Jinhua	119° 46'E, 29° 21'N
Honey-215	*Apis mellifera*	2015	Rape	processing facility	Jinhua	119° 46'E, 29° 21'N
Honey-216	*Apis mellifera*	2015	Rape	apiary	Jinhua	119° 46'E, 29° 21'N

Honey-217	*Apis mellifera*	2015	Citrus	apiary	Jinhua	119° 46'E, 29° 21'N
Honey-218	*Apis mellifera*	2015	Rape	apiary	Jinhua	119° 46'E, 29° 21'N
Honey-219	*Apis mellifera*	2015	Citrus	apiary	Jinhua	119° 46'E, 29° 21'N
Honey-220	*Apis mellifera*	2015	Citrus	apiary	Jinhua	119° 46'E, 29° 21'N
Honey-221	*Apis mellifera*	2015	Acacia	apiary	Jinhua	119° 46'E, 29° 21'N
Honey-222	*Apis mellifera*	2015	Linden	apiary	Jinhua	119° 46'E, 29° 21'N
Honey-223	*Apis mellifera*	2015	Rape	apiary	Jinhua	119° 46'E, 29° 21'N
Honey-224	*Apis mellifera*	2015	Acacia	processing facility	Shaoxing	120° 48'E, 30° 08'N
Honey-225	*Apis mellifera*	2015	Rape	apiary	Shaoxing	120° 48'E, 30° 08'N
Honey-226	*Apis mellifera*	2015	Acacia	processing facility	Shaoxing	120° 48'E, 30° 08'N
Honey-227	*Apis mellifera*	2015	Rape	processing facility	Shaoxing	120° 48'E, 30° 08'N
Honey-228	*Apis mellifera*	2015	Rape	processing facility	Shaoxing	120° 48'E, 30° 08'N
Honey-229	*Apis mellifera*	2015	Lucerne	apiary	Shaoxing	120° 48'E, 30° 08'N
Honey-230	*Apis mellifera*	2015	Rape	apiary	Shaoxing	120° 48'E, 30° 08'N
Honey-231	*Apis mellifera*	2015	Mutiflower	apiary	Shaoxing	120° 48'E, 30° 08'N
Honey-232	*Apis mellifera*	2015	Rape	apiary	Shaoxing	120° 48'E, 30° 08'N
Honey-233	*Apis mellifera*	2015	Rape	apiary	Shaoxing	120° 60'E, 30° 07'N
Honey-234	*Apis mellifera*	2015	Acacia	apiary	Shaoxing	120° 60'E, 30° 07'N
Honey-235	*Apis mellifera*	2015	Mutiflower	apiary	Shaoxing	120° 58'E, 30° 00'N
Honey-236	*Apis mellifera*	2016	Cherry	apiary	Hangzhou	119° 69'E, 29° 80'N
Honey-237	*Apis mellifera*	2016	Rape	apiary	Hangzhou	119° 69'E, 29° 80'N
Honey-238	*Apis mellifera*	2016	Mutiflower	apiary	Hangzhou	119° 69'E, 29° 80'N

Honey-239	*Apis mellifera*	2016	Rape	apiary	Hangzhou	119° 69'E, 29° 80'N
Honey-240	*Apis mellifera*	2016	Rape	apiary	Hangzhou	119° 69'E, 29° 80'N
Honey-241	*Apis mellifera*	2016	Rape	apiary	Hangzhou	119° 69'E, 29° 80'N
Honey-242	*Apis mellifera*	2016	Rape	apiary	Hangzhou	119° 69'E, 29° 80'N
Honey-243	*Apis mellifera*	2016	Rape	apiary	Hangzhou	119° 69'E, 29° 80'N
Honey-244	*Apis mellifera*	2016	Rape	apiary	Hangzhou	119° 69'E, 29° 80'N
Honey-245	*Apis mellifera*	2016	Mutiflower	apiary	Hangzhou	119° 69'E, 29° 80'N
Honey-246	*Apis mellifera*	2016	Motherwort	bee-product stores	Hangzhou	119° 69'E, 29° 80'N
Honey-247	*Apis mellifera*	2016	Mutiflower	bee-product stores	Hangzhou	119° 69'E, 29° 80'N
Honey-248	*Apis mellifera*	2016	Longan	supermarket	Hangzhou	119° 69'E, 29° 80'N
Honey-249	*Apis mellifera*	2016	Acacia	supermarket	Hangzhou	119° 69'E, 29° 80'N
Honey-250	*Apis mellifera*	2016	Acacia	supermarket	Hangzhou	119° 69'E, 29° 80'N
Honey-251	*Apis mellifera*	2016	Acacia	supermarket	Hangzhou	119° 69'E, 29° 80'N
Honey-252	*Apis mellifera*	2016	Acacia	bee-product stores	Quzhou	118° 63'E, 28° 74'N
Honey-253	*Apis cerana*	2016	Mutiflower	bee-product stores	Quzhou	118° 63'E, 28° 74'N
Honey-254	*Apis mellifera*	2016	Acacia	supermarket	Quzhou	118° 63'E, 28° 74'N
Honey-255	*Apis mellifera*	2016	Acacia	supermarket	Quzhou	118° 63'E, 28° 74'N
Honey-256	*Apis mellifera*	2016	Acacia	supermarket	Quzhou	118° 63'E, 28° 74'N
Honey-257	*Apis mellifera*	2016	Rape	apiary	Quzhou	118° 63'E, 28° 74'N
Honey-258	*Apis cerana*	2016	Sweet Osmanthus	apiary	Quzhou	118° 63'E, 28° 74'N
Honey-259	*Apis cerana*	2016	Mutiflower	apiary	Quzhou	118° 63'E, 28° 74'N
Honey-260	*Apis cerana*	2016	Mutiflower	apiary	Quzhou	118° 63'E, 28° 74'N

Honey-261	*Apis cerana*	2016	Mutiflower	apiary	Quzhou	118° 63'E, 28° 74'N
Honey-262	*Apis mellifera*	2016	Rape	apiary	Quzhou	118° 63'E, 28° 74'N
Honey-263	*Apis cerana*	2016	Mutiflower	apiary	Quzhou	118° 63'E, 28° 74'N
Honey-264	*Apis mellifera*	2016	Rape	apiary	Quzhou	118° 63'E, 28° 74'N
Honey-265	*Apis cerana*	2016	Mutiflower	apiary	Quzhou	118° 63'E, 28° 74'N
Honey-266	*Apis mellifera*	2016	Rape	apiary	Quzhou	118° 63'E, 28° 74'N
Honey-267	*Apis mellifera*	2016	Rape	apiary	Huzhou	119° 91'E, 30° 01'N
Honey-268	*Apis mellifera*	2016	Rape	apiary	Huzhou	119° 91'E, 30° 01'N
Honey-269	*Apis mellifera*	2016	Rape	apiary	Huzhou	119° 91'E, 30° 01'N
Honey-270	*Apis mellifera*	2016	Rape	apiary	Huzhou	119° 91'E, 30° 01'N
Honey-271	*Apis mellifera*	2016	Rape	apiary	Huzhou	119° 91'E, 30° 01'N
Honey-272	*Apis mellifera*	2016	Rape	apiary	Huzhou	119° 91'E, 30° 01'N
Honey-273	*Apis mellifera*	2016	Rape	apiary	Huzhou	119° 91'E, 30° 01'N
Honey-274	*Apis mellifera*	2016	Acacia	bee-product stores	Huzhou	119° 91'E, 30° 01'N
Honey-275	*Apis mellifera*	2016	Acacia	bee-product stores	Huzhou	119° 91'E, 30° 01'N
Honey-276	*Apis mellifera*	2016	Acacia	supermarket	Huzhou	119° 91'E, 30° 01'N
Honey-277	*Apis mellifera*	2016	Acacia	supermarket	Huzhou	119° 91'E, 30° 01'N
Honey-278	*Apis mellifera*	2016	Acacia	supermarket	Ningbo	121° 27'E, 30° 17'N
Honey-279	*Apis mellifera*	2016	Acacia	supermarket	Ningbo	121° 27'E, 30° 17'N
Honey-280	*Apis mellifera*	2016	Acacia	bee-product stores	Ningbo	121° 27'E, 30° 17'N
Honey-281	*Apis mellifera*	2016	Rape	apiary	Ningbo	121° 27'E, 30° 17'N
Honey-282	*Apis mellifera*	2016	Rape	apiary	Ningbo	121° 27'E, 30° 17'N

Honey-283	*Apis mellifera*	2016	Rape	apiary	Ningbo	121° 27'E, 30° 17'N
Honey-284	*Apis mellifera*	2016	Rape	apiary	Ningbo	121° 27'E, 30° 17'N
Honey-285	*Apis mellifera*	2016	Rape	apiary	Ningbo	121° 27'E, 30° 17'N
Honey-286	*Apis mellifera*	2016	Rape	apiary	Ningbo	121° 27'E, 30° 17'N
Honey-287	*Apis mellifera*	2016	Rape	apiary	Ningbo	121° 27'E, 30° 17'N
Honey-288	*Apis mellifera*	2016	Rape	apiary	Ningbo	121° 27'E, 30° 17'N
Honey-289	*Apis mellifera*	2016	Acacia	bee-product stores	Jinhua	119° 57'E, 29° 10'N
Honey-290	*Apis mellifera*	2016	Acacia	supermarket	Jinhua	119° 57'E, 29° 10'N
Honey-291	*Apis mellifera*	2016	Acacia	supermarket	Jinhua	119° 57'E, 29° 10'N
Honey-292	*Apis mellifera*	2016	Mutiflower	apiary	Jinhua	119° 57'E, 29° 10'N
Honey-293	*Apis mellifera*	2016	Mutiflower	apiary	Jinhua	119° 57'E, 29° 10'N
Honey-294	*Apis mellifera*	2016	Mutiflower	apiary	Jinhua	119° 57'E, 29° 10'N
Honey-295	*Apis mellifera*	2016	Mutiflower	apiary	Jinhua	119° 57'E, 29° 10'N
Honey-296	*Apis mellifera*	2016	Mutiflower	apiary	Jinhua	119° 57'E, 29° 10'N
Honey-297	*Apis mellifera*	2016	Mutiflower	apiary	Jinhua	119° 57'E, 29° 10'N
Honey-298	*Apis mellifera*	2016	Mutiflower	apiary	Jinhua	119° 57'E, 29° 10'N
Honey-299	*Apis mellifera*	2016	Mutiflower	apiary	Jinhua	119° 57'E, 29° 10'N
Honey-300	*Apis cerana*	2016	Mutiflower	apiary	Lishui	119° 91'E, 28° 45'N
Honey-301	*Apis cerana*	2016	Mutiflower	apiary	Lishui	119° 91'E, 28° 45'N
Honey-302	*Apis cerana*	2016	Mutiflower	apiary	Lishui	119° 91'E, 28° 45'N
Honey-303	*Apis cerana*	2016	Mutiflower	apiary	Lishui	119° 91'E, 28° 45'N
Honey-304	*Apis cerana*	2016	Mutiflower	apiary	Lishui	119° 91'E, 28° 45'N

Honey-305	*Apis cerana*	2016	Mutiflower	apiary	Lishui	119° 91'E, 28° 45'N
Honey-306	*Apis cerana*	2016	Mutiflower	apiary	Lishui	120° 09'E, 28° 66'N
Honey-307	*Apis cerana*	2016	Mutiflower	apiary	Lishui	120° 09'E, 28° 66'N
Honey-308	*Apis cerana*	2016	Mutiflower	apiary	Lishui	120° 09'E, 28° 66'N
Honey-309	*Apis cerana*	2016	Mutiflower	apiary	Lishui	120° 09'E, 28° 66'N
Honey-310	*Apis cerana*	2016	Mutiflower	apiary	Lishui	120° 09'E, 28° 66'N
Honey-311	*Apis mellifera*	2016	Acacia	processing facility	Lishui	120° 09'E, 28° 66'N
Honey-312	*Apis mellifera*	2016	Rape	processing facility	Lishui	120° 09'E, 28° 66'N
Honey-313	*Apis mellifera*	2016	Acacia	processing facility	Lishui	120° 09'E, 28° 66'N
Honey-314	*Apis mellifera*	2016	Rape	apiary	Lishui	120° 09'E, 28° 66'N
Honey-315	*Apis mellifera*	2016	Rape	apiary	Lishui	120° 09'E, 28° 66'N
Honey-316	*Apis mellifera*	2016	Rape	apiary	Lishui	120° 09'E, 28° 66'N
Honey-317	*Apis mellifera*	2016	Rape	apiary	Lishui	119° 48'E, 28° 45'N
Honey-318	*Apis mellifera*	2016	Rape	apiary	Lishui	119° 48'E, 28° 45'N
Honey-319	*Apis mellifera*	2016	Rape	apiary	Lishui	119° 48'E, 28° 45'N
Honey-320	*Apis mellifera*	2016	Rape	apiary	Lishui	119° 48'E, 28° 45'N
Honey-321	*Apis mellifera*	2016	Acacia	supermarket	Lishui	119° 48'E, 28° 45'N
Honey-322	*Apis mellifera*	2016	Sweet Osmanthus	supermarket	Lishui	119° 48'E, 28° 45'N
Honey-323	*Apis cerana*	2016	Mutiflower	apiary	Lishui	119° 28'E, 28° 59'N
Honey-324	*Apis cerana*	2016	Mutiflower	apiary	Lishui	119° 28'E, 28° 59'N
Honey-325	*Apis cerana*	2016	Mutiflower	apiary	Lishui	119° 28'E, 28° 59'N
Honey-326	*Apis cerana*	2016	Mutiflower	apiary	Lishui	119° 28'E, 28° 59'N

Honey-327	*Apis cerana*	2016	Mutiflower	apiary	Lishui	119° 28'E, 28° 59'N
Honey-328	*Apis cerana*	2016	Mutiflower	apiary	Lishui	119° 28'E, 28° 59'N
Honey-329	*Apis cerana*	2016	Mutiflower	apiary	Lishui	119° 28'E, 28° 59'N
Honey-330	*Apis cerana*	2016	Mutiflower	apiary	Lishui	119° 28'E, 28° 59'N
Honey-331	*Apis cerana*	2016	Mutiflower	apiary	Lishui	119° 28'E, 28° 59'N
Honey-332	*Apis cerana*	2016	Mutiflower	apiary	Lishui	119° 28'E, 28° 59'N
Honey-333	*Apis cerana*	2016	Mutiflower	apiary	Lishui	119° 28'E, 28° 59'N
Honey-334	*Apis cerana*	2016	Mutiflower	apiary	Lishui	119° 28'E, 28° 59'N
Honey-335	*Apis cerana*	2016	Mutiflower	bee-product stores	Lishui	119° 28'E, 28° 59'N
Honey-336	*Apis mellifera*	2016	Acacia	supermarket	Hangzhou	119° 28'E, 28° 48'N
Honey-337	*Apis mellifera*	2016	Acacia	supermarket	Hangzhou	119° 28'E, 28° 48'N
Honey-338	*Apis mellifera*	2016	Acacia	supermarket	Hangzhou	119° 28'E, 28° 48'N
Honey-339	*Apis mellifera*	2016	Acacia	apiary	Hangzhou	119° 28'E, 28° 48'N
Honey-340	*Apis mellifera*	2016	Lychee	supermarket	Hangzhou	119° 28'E, 28° 48'N
Honey-341	*Apis mellifera*	2016	Mutiflower	apiary	Hangzhou	119° 28'E, 28° 48'N
Honey-342	*Apis mellifera*	2016	Mutiflower	apiary	Hangzhou	119° 28'E, 28° 48'N
Honey-343	*Apis mellifera*	2016	Mutiflower	apiary	Hangzhou	119° 28'E, 28° 48'N
Honey-344	*Apis mellifera*	2016	Mutiflower	apiary	Hangzhou	119° 28'E, 28° 48'N
Honey-345	*Apis mellifera*	2016	Mutiflower	apiary	Hangzhou	119° 28'E, 28° 48'N
Honey-346	*Apis mellifera*	2016	Mutiflower	apiary	Hangzhou	119° 28'E, 28° 48'N
Honey-347	*Apis mellifera*	2016	Mutiflower	apiary	Hangzhou	119° 28'E, 28° 48'N
Honey-348	*Apis mellifera*	2016	Mutiflower	apiary	Hangzhou	119° 28'E, 28° 48'N

Honey-349	*Apis mellifera*	2016	Mutiflower	apiary	Hangzhou	119° 28'E, 28° 48'N
Honey-350	*Apis mellifera*	2016	Mutiflower	apiary	Hangzhou	119° 28'E, 28° 48'N
Honey-351	*Apis cerana*	2017	Rape	apiary	Quzhou	118° 63'E, 28° 74'N
Honey-352	*Apis cerana*	2017	Mutiflower	apiary	Quzhou	118° 63'E, 28° 74'N
Honey-353	*Apis cerana*	2017	Mutiflower	apiary	Quzhou	118° 63'E, 28° 74'N
Honey-354	*Apis cerana*	2017	Mutiflower	apiary	Quzhou	118° 63'E, 28° 74'N
Honey-355	*Apis cerana*	2017	Rape	bee-product stores	Quzhou	118° 63'E, 28° 74'N
Honey-356	*Apis mellifera*	2017	Longan	supermarket	Quzhou	118° 63'E, 28° 74'N
Honey-357	*Apis mellifera*	2017	Rape	apiary	Quzhou	118° 63'E, 28° 74'N
Honey-358	*Apis cerana*	2017	Citrus	apiary	Quzhou	118° 63'E, 28° 74'N
Honey-359	*Apis cerana*	2017	Mutiflower	apiary	Quzhou	118° 63'E, 28° 74'N
Honey-360	*Apis cerana*	2017	Citrus	apiary	Quzhou	118° 63'E, 28° 74'N
Honey-361	*Apis cerana*	2017	Rape	apiary	Quzhou	118° 63'E, 28° 74'N
Honey-362	*Apis cerana*	2017	Mutiflower	apiary	Quzhou	118° 63'E, 28° 74'N
Honey-363	*Apis mellifera*	2017	Rape	apiary	Huzhou	119° 91'E, 30° 01'N
Honey-364	*Apis mellifera*	2017	Rape	apiary	Huzhou	119° 91'E, 30° 01'N
Honey-365	*Apis mellifera*	2017	Rape	apiary	Huzhou	119° 91'E, 30° 01'N
Honey-366	*Apis mellifera*	2017	Rape	apiary	Huzhou	119° 91'E, 30° 01'N
Honey-367	*Apis mellifera*	2017	Rape	apiary	Huzhou	119° 91'E, 30° 01'N
Honey-368	*Apis mellifera*	2017	Rape	apiary	Huzhou	119° 91'E, 30° 01'N
Honey-369	*Apis mellifera*	2017	Rape	apiary	Huzhou	119° 91'E, 30° 01'N
Honey-370	*Apis mellifera*	2017	Rape	apiary	Huzhou	119° 91'E, 30° 01'N

Honey-371	*Apis mellifera*	2017	Rape	apiary	Huzhou	119° 91'E, 30° 01'N
Honey-372	*Apis mellifera*	2017	Rape	apiary	Huzhou	119° 91'E, 30° 01'N
Honey-373	*Apis mellifera*	2017	Acacia	supermarket	Huzhou	119° 91'E, 30° 01'N
Honey-374	*Apis mellifera*	2017	Mutiflower	bee-product stores	Huzhou	119° 91'E, 30° 01'N
Honey-375	*Apis mellifera*	2017	Rape	apiary	Ningbo	121° 27'E, 30° 17'N
Honey-376	*Apis mellifera*	2017	Mutiflower	apiary	Ningbo	121° 27'E, 30° 17'N
Honey-377	*Apis mellifera*	2017	Rape	apiary	Ningbo	121° 27'E, 30° 17'N
Honey-378	*Apis mellifera*	2017	Mutiflower	apiary	Ningbo	121° 27'E, 30° 17'N
Honey-379	*Apis mellifera*	2017	Rape	apiary	Ningbo	121° 27'E, 30° 17'N
Honey-380	*Apis mellifera*	2017	Mutiflower	apiary	Ningbo	121° 27'E, 30° 17'N
Honey-381	*Apis mellifera*	2017	Rape	apiary	Ningbo	121° 27'E, 30° 17'N
Honey-382	*Apis mellifera*	2017	Rape	apiary	Ningbo	121° 27'E, 30° 17'N
Honey-383	*Apis mellifera*	2017	Rape	apiary	Ningbo	121° 27'E, 30° 17'N
Honey-384	*Apis mellifera*	2017	Rape	apiary	Ningbo	121° 27'E, 30° 17'N
Honey-385	*Apis mellifera*	2017	Acacia	supermarket	Ningbo	121° 27'E, 30° 17'N
Honey-386	*Apis mellifera*	2017	Acacia	bee-product stores	Ningbo	121° 27'E, 30° 17'N
Honey-387	*Apis cerana*	2017	Mutiflower	apiary	Ningbo	121° 27'E, 30° 17'N
Honey-388	*Apis cerana*	2017	Longan	apiary	Jinhua	119° 57'E, 29° 10'N
Honey-389	*Apis mellifera*	2017	Rape	apiary	Jinhua	119° 57'E, 29° 10'N
Honey-390	*Apis mellifera*	2017	Citrus	apiary	Jinhua	119° 57'E, 29° 10'N
Honey-391	*Apis cerana*	2017	Sweet Osmanthus	bee-product stores	Jinhua	119° 57'E, 29° 10'N
Honey-392	*Apis mellifera*	2017	Acacia	supermarket	Jinhua	119° 57'E, 29° 10'N

Honey-393	*Apis cerana*	2017	Mutiflower	apiary	Jinhua	119° 57'E, 29° 10'N
Honey-394	*Apis cerana*	2017	Longan	apiary	Jinhua	119° 57'E, 29° 10'N
Honey-395	*Apis cerana*	2017	Mutiflower	apiary	Jinhua	119° 57'E, 29° 10'N
Honey-396	*Apis cerana*	2017	Mutiflower	apiary	Jinhua	119° 57'E, 29° 10'N
Honey-397	*Apis cerana*	2017	Rape	apiary	Jinhua	119° 57'E, 29° 10'N
Honey-398	*Apis cerana*	2017	Mutiflower	apiary	Jinhua	119° 57'E, 29° 10'N
Honey-399	*Apis cerana*	2017	Mutiflower	apiary	Jinhua	119° 57'E, 29° 10'N
Honey-400	*Apis cerana*	2017	Longan	apiary	Jinhua	119° 46'E, 29° 21'N
Honey-401	*Apis cerana*	2017	Mutiflower	apiary	Jinhua	119° 46'E, 29° 21'N
Honey-402	*Apis cerana*	2017	Mutiflower	apiary	Jinhua	119° 46'E, 29° 21'N
Honey-403	*Apis mellifera*	2017	Acacia	supermarket	Jinhua	119° 46'E, 29° 21'N
Honey-404	*Apis mellifera*	2017	Longan	apiary	Jinhua	119° 46'E, 29° 21'N
Honey-405	*Apis mellifera*	2017	Citrus	apiary	Jinhua	119° 46'E, 29° 21'N
Honey-406	*Apis cerana*	2017	Longan	apiary	Jinhua	119° 46'E, 29° 21'N
Honey-407	*Apis cerana*	2017	Mutiflower	apiary	Jinhua	119° 46'E, 29° 21'N
Honey-408	*Apis cerana*	2017	Mutiflower	apiary	Jinhua	119° 46'E, 29° 21'N
Honey-409	*Apis cerana*	2017	Citrus	apiary	Jinhua	119° 46'E, 29° 21'N
Honey-410	*Apis cerana*	2017	Longan	apiary	Jinhua	119° 46'E, 29° 21'N
Honey-411	*Apis cerana*	2017	Mutiflower	apiary	Jinhua	119° 46'E, 29° 21'N
Honey-412	*Apis cerana*	2017	Mutiflower	apiary	Lishui	119° 28'E, 28° 59'N
Honey-413	*Apis cerana*	2017	Rape	apiary	Lishui	119° 28'E, 28° 59'N
Honey-414	*Apis cerana*	2017	Citrus	apiary	Lishui	119° 28'E, 28° 59'N

Honey-415	*Apis cerana*	2017	Mutiflower	apiary	Lishui	119° 28'E, 28° 59'N
Honey-416	*Apis cerana*	2017	Chestnut	apiary	Lishui	119° 28'E, 28° 59'N
Honey-417	*Apis cerana*	2017	Mutiflower	apiary	Lishui	119° 28'E, 28° 59'N
Honey-418	*Apis cerana*	2017	Mutiflower	apiary	Lishui	119° 28'E, 28° 59'N
Honey-419	*Apis cerana*	2017	Rape	apiary	Lishui	119° 28'E, 28° 59'N
Honey-420	*Apis cerana*	2017	Mutiflower	bee-product stores	Lishui	119° 28'E, 28° 59'N
Honey-421	*Apis cerana*	2017	Rape	bee-product stores	Lishui	119° 28'E, 28° 59'N
Honey-422	*Apis cerana*	2017	Mutiflower	apiary	Lishui	119° 28'E, 28° 59'N
Honey-423	*Apis mellifera*	2017	Acacia	supermarket	Lishui	119° 28'E, 28° 59'N
Honey-424	*Apis mellifera*	2017	Jujube	supermarket	Lishui	119° 28'E, 28° 59'N
Honey-425	*Apis cerana*	2017	Chestnut	apiary	Lishui	119° 28'E, 28° 59'N
Honey-426	*Apis cerana*	2017	Mutiflower	apiary	Lishui	120° 09'E, 28° 66'N
Honey-427	*Apis cerana*	2017	Mutiflower	apiary	Lishui	120° 09'E, 28° 66'N
Honey-428	*Apis cerana*	2017	Mutiflower	apiary	Lishui	120° 09'E, 28° 66'N
Honey-429	*Apis cerana*	2017	Mutiflower	apiary	Lishui	120° 09'E, 28° 66'N
Honey-430	*Apis mellifera*	2017	Acacia	apiary	Lishui	120° 09'E, 28° 66'N
Honey-431	*Apis cerana*	2017	Mutiflower	apiary	Lishui	120° 09'E, 28° 66'N
Honey-432	*Apis cerana*	2017	Mutiflower	apiary	Lishui	120° 09'E, 28° 66'N
Honey-433	*Apis cerana*	2017	Mutiflower	apiary	Lishui	120° 09'E, 28° 66'N
Honey-434	*Apis mellifera*	2017	Rape	apiary	Lishui	120° 09'E, 28° 66'N
Honey-435	*Apis mellifera*	2017	Linden	apiary	Lishui	120° 09'E, 28° 66'N
Honey-436	*Apis mellifera*	2017	Acacia	bee-product stores	Lishui	120° 09'E, 28° 66'N

Honey-437	*Apis mellifera*	2017	Jujube	bee-product stores	Lishui	120° 09'E, 28° 66'N
Honey-438	*Apis mellifera*	2017	Longan	supermarket	Lishui	120° 09'E, 28° 66'N
Honey-439	*Apis mellifera*	2017	Mutiflower	supermarket	Lishui	120° 09'E, 28° 66'N
Honey-440	*Apis mellifera*	2017	Chaste	processing facility	Quzhou	118° 63'E, 28° 74'N
Honey-441	*Apis cerana*	2017	Mutiflower	apiary	Quzhou	118° 63'E, 28° 74'N
Honey-442	*Apis mellifera*	2017	Rape	apiary	Quzhou	118° 63'E, 28° 74'N
Honey-443	*Apis mellifera*	2017	Acacia	processing facility	Quzhou	118° 63'E, 28° 74'N
Honey-444	*Apis mellifera*	2017	Rape	apiary	Quzhou	118° 63'E, 28° 74'N
Honey-445	*Apis mellifera*	2017	Rape	apiary	Quzhou	118° 63'E, 28° 74'N
Honey-446	*Apis mellifera*	2017	Chaste	processing facility	Quzhou	118° 63'E, 28° 74'N
Honey-447	*Apis mellifera*	2017	Chaste	processing facility	Quzhou	118° 63'E, 28° 74'N
Honey-448	*Apis mellifera*	2017	Mutiflower	apiary	Quzhou	118° 63'E, 28° 74'N
Honey-449	*Apis mellifera*	2017	Mutiflower	apiary	Quzhou	118° 63'E, 28° 74'N
Honey-450	*Apis mellifera*	2017	Rape	apiary	Quzhou	118° 63'E, 28° 74'N
Honey-451	*Apis mellifera*	2017	Rape	apiary	Quzhou	118° 63'E, 28° 74'N
Honey-452	*Apis mellifera*	2017	Acacia	supermarket	Quzhou	118° 63'E, 28° 74'N
Honey-453	*Apis cerana*	2017	Mutiflower	apiary	Lishui	120° 09'E, 28° 66'N
Honey-454	*Apis cerana*	2017	Mutiflower	apiary	Lishui	120° 09'E, 28° 66'N
Honey-455	*Apis cerana*	2017	Mutiflower	apiary	Lishui	120° 09'E, 28° 66'N
Honey-456	*Apis cerana*	2017	Mutiflower	apiary	Lishui	120° 09'E, 28° 66'N
Honey-457	*Apis cerana*	2017	Mutiflower	apiary	Lishui	120° 09'E, 28° 66'N
Honey-458	*Apis cerana*	2017	Mutiflower	apiary	Lishui	120° 09'E, 28° 66'N

Honey-459	*Apis cerana*	2017	Mutiflower	apiary	Lishui	120° 09'E, 28° 66'N
Honey-460	*Apis mellifera*	2017	Acacia	supermarket	Lishui	120° 09'E, 28° 66'N
Honey-461	*Apis mellifera*	2017	Acacia	processing facility	Lishui	120° 09'E, 28° 66'N
Honey-462	*Apis mellifera*	2017	Acacia	processing facility	Lishui	120° 09'E, 28° 66'N
Honey-463	*Apis mellifera*	2017	Acacia	processing facility	Lishui	120° 09'E, 28° 66'N
Honey-464	*Apis mellifera*	2017	Acacia	processing facility	Lishui	120° 09'E, 28° 66'N
Honey-465	*Apis mellifera*	2017	Linden	bee-product stores	Huzhou	119° 91'E, 30° 01'N
Honey-466	*Apis mellifera*	2017	Acacia	bee-product stores	Huzhou	119° 91'E, 30° 01'N
Honey-467	*Apis mellifera*	2017	Rape	bee-product stores	Huzhou	119° 91'E, 30° 01'N
Honey-468	*Apis mellifera*	2017	Acacia	bee-product stores	Huzhou	119° 91'E, 30° 01'N
Honey-469	*Apis mellifera*	2017	Acacia	supermarket	Huzhou	119° 91'E, 30° 01'N
Honey-470	*Apis mellifera*	2017	Rape	apiary	Huzhou	119° 91'E, 30° 01'N
Honey-471	*Apis mellifera*	2017	Rape	apiary	Huzhou	119° 91'E, 30° 01'N
Honey-472	*Apis mellifera*	2017	Rape	apiary	Huzhou	119° 91'E, 30° 01'N
Honey-473	*Apis mellifera*	2017	Rape	apiary	Huzhou	119° 91'E, 30° 01'N
Honey-474	*Apis cerana*	2017	Rape	apiary	Huzhou	119° 91'E, 30° 01'N
Honey-475	*Apis mellifera*	2017	Rape	apiary	Huzhou	119° 91'E, 30° 01'N
Honey-476	*Apis mellifera*	2017	Rape	apiary	Huzhou	119° 91'E, 30° 01'N
Honey-477	*Apis mellifera*	2017	Rape	apiary	Huzhou	119° 91'E, 30° 01'N
Honey-478	*Apis mellifera*	2017	Acacia	apiary	Ningbo	121° 27'E, 30° 17'N
Honey-479	*Apis mellifera*	2017	Quillaja saponaria	apiary	Ningbo	121° 27'E, 30° 17'N
Honey-480	*Apis mellifera*	2017	Acacia	processing facility	Ningbo	121° 27'E, 30° 17'N

Honey-481	*Apis mellifera*	2017	Mutiflower	processing facility	Ningbo	121° 27'E, 30° 17'N
Honey-482	*Apis mellifera*	2017	Rape	apiary	Ningbo	121° 27'E, 30° 17'N
Honey-483	*Apis mellifera*	2017	Acacia	apiary	Ningbo	121° 27'E, 30° 17'N
Honey-484	*Apis mellifera*	2017	Mutiflower	apiary	Ningbo	121° 27'E, 30° 17'N
Honey-485	*Apis mellifera*	2017	Acacia	apiary	Ningbo	121° 27'E, 30° 17'N
Honey-486	*Apis mellifera*	2017	Rape	apiary	Ningbo	121° 27'E, 30° 17'N
Honey-487	*Apis mellifera*	2017	Acacia	apiary	Ningbo	121° 27'E, 30° 17'N
Honey-488	*Apis mellifera*	2017	Acacia	processing facility	Ningbo	121° 27'E, 30° 17'N
Honey-489	*Apis mellifera*	2017	Acacia	supermarket	Ningbo	121° 27'E, 30° 17'N
Honey-490	*Apis mellifera*	2017	Acacia	bee-product stores	Ningbo	121° 27'E, 30° 17'N
Honey-491	*Apis mellifera*	2017	Acacia	bee-product stores	Hangzhou	119° 69'E, 29° 80'N
Honey-492	*Apis mellifera*	2017	Rape	apiary	Hangzhou	119° 69'E, 29° 80'N
Honey-493	*Apis mellifera*	2017	Chaste	apiary	Hangzhou	119° 69'E, 29° 80'N
Honey-494	*Apis mellifera*	2017	Acacia	apiary	Hangzhou	119° 69'E, 29° 80'N
Honey-495	*Apis mellifera*	2017	Acacia	apiary	Hangzhou	119° 69'E, 29° 80'N
Honey-496	*Apis mellifera*	2017	Linden	apiary	Hangzhou	119° 69'E, 29° 80'N
Honey-497	*Apis mellifera*	2017	Acacia	apiary	Hangzhou	119° 69'E, 29° 80'N
Honey-498	*Apis mellifera*	2017	Linden	apiary	Hangzhou	119° 69'E, 29° 80'N
Honey-499	*Apis mellifera*	2017	Acacia	apiary	Hangzhou	119° 69'E, 29° 80'N
Honey-500	*Apis mellifera*	2017	Rape	bee-product stores	Hangzhou	119° 69'E, 29° 80'N
Honey-501	*Apis mellifera*	2017	Citrus	bee-product stores	Hangzhou	119° 69'E, 29° 80'N
Honey-502	*Apis mellifera*	2017	Acacia	bee-product stores	Hangzhou	119° 69'E, 29° 80'N

Honey-503	*Apis mellifera*	2017	Mutiflower	supermarket	Hangzhou	119° 69'E, 29° 80'N
Honey-504	*Apis cerana*	2017	Mutiflower	apiary	Lishui	119° 28'E, 28° 59'N
Honey-505	*Apis cerana*	2017	Mutiflower	apiary	Lishui	119° 28'E, 28° 59'N
Honey-506	*Apis cerana*	2017	Mutiflower	apiary	Lishui	119° 28'E, 28° 59'N
Honey-507	*Apis cerana*	2017	Mutiflower	apiary	Lishui	119° 28'E, 28° 59'N
Honey-508	*Apis cerana*	2017	Mutiflower	apiary	Lishui	119° 28'E, 28° 59'N
Honey-509	*Apis mellifera*	2017	Linden	supermarket	Lishui	119° 28'E, 28° 59'N
Honey-510	*Apis cerana*	2017	Mutiflower	bee-product stores	Lishui	119° 28'E, 28° 59'N
Honey-511	*Apis cerana*	2017	Mutiflower	bee-product stores	Lishui	119° 28'E, 28° 59'N
Honey-512	*Apis cerana*	2017	Mutiflower	apiary	Lishui	119° 28'E, 28° 59'N
Honey-513	*Apis cerana*	2017	Mutiflower	apiary	Lishui	119° 28'E, 28° 59'N
Honey-514	*Apis cerana*	2017	Mutiflower	apiary	Lishui	119° 28'E, 28° 59'N
Honey-515	*Apis cerana*	2017	Mutiflower	bee-product stores	Lishui	119° 28'E, 28° 59'N
Honey-516	*Apis mellifera*	2017	Longan	processing facility	Jinhua	119° 46'E, 29° 21'N
Honey-517	*Apis mellifera*	2017	China Soapberry	processing facility	Jinhua	119° 46'E, 29° 21'N
Honey-518	*Apis cerana*	2017	Mutiflower	apiary	Jinhua	119° 46'E, 29° 21'N
Honey-519	*Apis cerana*	2017	Mutiflower	apiary	Jinhua	119° 46'E, 29° 21'N
Honey-520	*Apis cerana*	2017	Mutiflower	apiary	Jinhua	119° 46'E, 29° 21'N
Honey-521	*Apis cerana*	2017	Mutiflower	apiary	Jinhua	119° 46'E, 29° 21'N
Honey-522	*Apis mellifera*	2017	Mutiflower	apiary	Jinhua	119° 46'E, 29° 21'N
Honey-523	*Apis mellifera*	2017	Acacia	apiary	Jinhua	119° 46'E, 29° 21'N
Honey-524	*Apis mellifera*	2017	Jujube	apiary	Jinhua	119° 46'E, 29° 21'N

Honey-525	*Apis mellifera*	2017	Acacia	supermarket	Jinhua	119° 46'E, 29° 21'N
Honey-526	*Apis mellifera*	2017	Acacia	supermarket	Jinhua	119° 46'E, 29° 21'N
Honey-527	*Apis mellifera*	2017	Acacia	bee-product stores	Jinhua	119° 46'E, 29° 21'N
Honey-528	*Apis mellifera*	2017	Mutiflower	apiary	Jinhua	119° 57'E, 29° 10'N
Honey-529	*Apis mellifera*	2017	Mutiflower	apiary	Jinhua	119° 57'E, 29° 10'N
Honey-530	*Apis mellifera*	2017	Mutiflower	apiary	Jinhua	119° 57'E, 29° 10'N
Honey-531	*Apis mellifera*	2017	Rape	apiary	Jinhua	119° 57'E, 29° 10'N
Honey-532	*Apis mellifera*	2017	China Soapberry	apiary	Jinhua	119° 57'E, 29° 10'N
Honey-533	*Apis mellifera*	2017	Mutiflower	apiary	Jinhua	119° 57'E, 29° 10'N
Honey-534	*Apis mellifera*	2017	Longan	apiary	Jinhua	119° 57'E, 29° 10'N
Honey-535	*Apis mellifera*	2017	Acacia	bee-product stores	Jinhua	119° 57'E, 29° 10'N
Honey-536	*Apis mellifera*	2017	Linden	bee-product stores	Jinhua	119° 57'E, 29° 10'N
Honey-537	*Apis mellifera*	2017	Jujube	bee-product stores	Jinhua	119° 57'E, 29° 10'N
Honey-538	*Apis mellifera*	2017	Acacia	supermarket	Jinhua	119° 57'E, 29° 10'N
Honey-539	*Apis mellifera*	2017	Acacia	supermarket	Jinhua	119° 57'E, 29° 10'N
Honey-540	*Apis mellifera*	2018	Mutiflower	apiary	Quzhou	118° 63'E, 28° 74'N
Honey-541	*Apis mellifera*	2018	Rape	apiary	Quzhou	118° 63'E, 28° 74'N
Honey-542	*Apis mellifera*	2018	Mutiflower	apiary	Quzhou	118° 63'E, 28° 74'N
Honey-543	*Apis mellifera*	2018	Mutiflower	apiary	Quzhou	118° 63'E, 28° 74'N
Honey-544	*Apis mellifera*	2018	Mutiflower	apiary	Quzhou	118° 63'E, 28° 74'N
Honey-545	*Apis mellifera*	2018	Mutiflower	apiary	Quzhou	118° 63'E, 28° 74'N
Honey-546	*Apis mellifera*	2018	Mutiflower	apiary	Quzhou	118° 63'E, 28° 74'N

Honey-547	*Apis mellifera*	2018	Mutiflower	apiary	Quzhou	118° 63'E, 28° 74'N
Honey-548	*Apis mellifera*	2018	Mutiflower	apiary	Quzhou	118° 63'E, 28° 74'N
Honey-549	*Apis mellifera*	2018	Longan	apiary	Hangzhou	119° 69'E, 29° 80'N
Honey-550	*Apis mellifera*	2018	Rape	apiary	Hangzhou	119° 69'E, 29° 80'N
Honey-551	*Apis mellifera*	2018	Rape	apiary	Shaoxing	120° 48'E, 30° 08'N
Honey-552	*Apis mellifera*	2018	Rape	apiary	Shaoxing	120° 48'E, 30° 08'N
Honey-553	*Apis mellifera*	2018	Rape	apiary	Shaoxing	120° 48'E, 30° 08'N
Honey-554	*Apis mellifera*	2018	Rape	apiary	Shaoxing	120° 48'E, 30° 08'N
Honey-555	*Apis mellifera*	2018	Rape	apiary	Shaoxing	120° 48'E, 30° 08'N
Honey-556	*Apis mellifera*	2018	Rape	apiary	Shaoxing	120° 48'E, 30° 08'N
Honey-557	*Apis mellifera*	2018	Rape	apiary	Shaoxing	120° 48'E, 30° 08'N
Honey-558	*Apis mellifera*	2018	Rape	apiary	Shaoxing	120° 48'E, 30° 08'N
Honey-559	*Apis mellifera*	2018	Rape	apiary	Shaoxing	120° 48'E, 30° 08'N
Honey-560	*Apis mellifera*	2018	Rape	apiary	Huzhou	119° 91'E, 30° 01'N
Honey-561	*Apis cerana*	2018	Mutiflower	apiary	Huzhou	119° 91'E, 30° 01'N
Honey-562	*Apis mellifera*	2018	Rape	apiary	Huzhou	119° 91'E, 30° 01'N
Honey-563	*Apis mellifera*	2018	Rape	apiary	Huzhou	119° 91'E, 30° 01'N
Honey-564	*Apis mellifera*	2018	Rape	apiary	Huzhou	119° 91'E, 30° 01'N
Honey-565	*Apis mellifera*	2018	Mutiflower	apiary	Jinhua	119° 57'E, 29° 10'N
Honey-566	*Apis mellifera*	2018	Longan	apiary	Jinhua	119° 57'E, 29° 10'N
Honey-567	*Apis mellifera*	2018	Longan	apiary	Jinhua	119° 57'E, 29° 10'N
Honey-568	*Apis mellifera*	2018	Mutiflower	apiary	Jinhua	119° 57'E, 29° 10'N

Honey-569	*Apis mellifera*	2018	Mutiflower	apiary	Jinhua	119° 57'E, 29° 10'N
Honey-570	*Apis mellifera*	2018	Mutiflower	apiary	Jinhua	119° 57'E, 29° 10'N
Honey-571	*Apis mellifera*	2018	Mutiflower	apiary	Jinhua	119° 57'E, 29° 10'N
Honey-572	*Apis mellifera*	2018	Mutiflower	apiary	Jinhua	119° 69'E, 29° 10'N
Honey-573	*Apis mellifera*	2018	Rape	apiary	Jinhua	119° 57'E, 29° 10'N
Honey-574	*Apis mellifera*	2018	Mutiflower	processing facility	Jinhua	119° 46'E, 29° 21'N
Honey-575	*Apis mellifera*	2018	Mutiflower	apiary	Jinhua	119° 46'E, 29° 21'N
Honey-576	*Apis mellifera*	2018	Rape	apiary	Jinhua	119° 46'E, 29° 21'N
Honey-577	*Apis mellifera*	2018	Sweet Osmanthus	apiary	Jinhua	119° 46'E, 29° 21'N
Honey-578	*Apis mellifera*	2018	Citrus	apiary	Jinhua	119° 46'E, 29° 21'N
Honey-579	*Apis mellifera*	2018	Longan	apiary	Jinhua	119° 46'E, 29° 21'N
Honey-580	*Apis mellifera*	2018	Rape	apiary	Jinhua	119° 46'E, 29° 21'N
Honey-581	*Apis mellifera*	2018	Rape	apiary	Ningbo	121° 27'E, 30° 17'N
Honey-582	*Apis mellifera*	2018	Rape	apiary	Ningbo	121° 27'E, 30° 17'N
Honey-583	*Apis mellifera*	2018	Rape	apiary	Ningbo	121° 27'E, 30° 17'N
Honey-584	*Apis mellifera*	2018	Rape	apiary	Ningbo	121° 27'E, 30° 17'N
Honey-585	*Apis mellifera*	2018	Rape	apiary	Ningbo	121° 27'E, 30° 17'N
Honey-586	*Apis mellifera*	2018	Rape	apiary	Ningbo	121° 27'E, 30° 17'N
Honey-587	*Apis mellifera*	2018	Rape	apiary	Ningbo	121° 27'E, 30° 17'N
Honey-588	*Apis mellifera*	2018	Motherwort	processing facility	Ningbo	121° 27'E, 30° 17'N
Honey-589	*Apis cerana*	2018	Rape	apiary	Lishui	120° 09'E, 28° 66'N
Honey-590	*Apis cerana*	2018	Rape	apiary	Lishui	120° 09'E, 28° 66'N

Honey-591	*Apis cerana*	2018	Rape	apiary	Lishui	120° 09'E, 28° 66'N
Honey-592	*Apis cerana*	2018	Rape	apiary	Lishui	120° 09'E, 28° 66'N
Honey-593	*Apis cerana*	2018	Rape	apiary	Lishui	120° 09'E, 28° 66'N
Honey-594	*Apis cerana*	2018	Rape	apiary	Lishui	120° 09'E, 28° 66'N
Honey-595	*Apis cerana*	2018	Rape	apiary	Lishui	120° 09'E, 28° 66'N
Honey-596	*Apis cerana*	2018	Rape	apiary	Lishui	120° 09'E, 28° 66'N
Honey-597	*Apis cerana*	2018	Rape	apiary	Lishui	120° 09'E, 28° 66'N
Honey-598	*Apis cerana*	2018	Rape	apiary	Lishui	119° 28'E, 28° 59'N
Honey-599	*Apis cerana*	2018	Rape	apiary	Lishui	119° 28'E, 28° 59'N
Honey-600	*Apis cerana*	2018	Rape	apiary	Lishui	119° 28'E, 28° 59'N
Honey-601	*Apis cerana*	2018	Rape	apiary	Lishui	119° 28'E, 28° 59'N
Honey-602	*Apis cerana*	2018	Mutiflower	processing facility	Shaoxing	120° 48'E, 30° 08'N
Honey-603	*Apis cerana*	2018	Mutiflower	processing facility	Shaoxing	120° 48'E, 30° 08'N
Honey-604	*Apis mellifera*	2018	Chinese milk vetch	processing facility	Ningbo	121° 27'E, 30° 17'N
Honey-605	*Apis mellifera*	2018	Chinese milk vetch	processing facility	Ningbo	121° 27'E, 30° 17'N
Honey-606	*Apis cerana*	2018	Mutiflower	processing facility	Quzhou	118° 63'E, 28° 74'N
Honey-607	*Apis cerana*	2018	Mutiflower	processing facility	Quzhou	118° 63'E, 28° 74'N
Honey-608	*Apis mellifera*	2018	Longan	processing facility	Quzhou	118° 63'E, 28° 74'N
Honey-609	*Apis mellifera*	2018	Longan	processing facility	Quzhou	118° 63'E, 28° 74'N
Honey-610	*Apis mellifera*	2018	Jujube	apiary	Jinhua	119° 46'E, 29° 21'N
Honey-611	*Apis mellifera*	2018	Mutiflower	apiary	Jinhua	119° 46'E, 29° 21'N
Honey-612	*Apis mellifera*	2018	Mutiflower	apiary	Shaoxing	120° 49'E, 30° 09'N

Honey-613	*Apis mellifera*	2018	Mutiflower	apiary	Shaoxing	120° 49'E, 30° 09'N
Honey-614	*Apis mellifera*	2018	Mutiflower	apiary	Ningbo	121° 15'E, 30° 04'N
Honey-615	*Apis mellifera*	2018	Mutiflower	apiary	Ningbo	121° 27'E, 30° 17'N
Honey-616	*Apis mellifera*	2018	Mutiflower	apiary	Ningbo	121° 27'E, 30° 17'N
Honey-617	*Apis mellifera*	2018	Mutiflower	apiary	Ningbo	121° 27'E, 30° 17'N
Honey-618	*Apis mellifera*	2018	Mutiflower	apiary	Ningbo	121° 27'E, 30° 17'N
Honey-619	*Apis mellifera*	2018	Mutiflower	apiary	Ningbo	121° 87'E, 29° 48'N
Honey-620	*Apis mellifera*	2018	Mutiflower	apiary	Ningbo	121° 87'E, 29° 48'N
Honey-621	*Apis cerana*	2018	Mutiflower	apiary	Hangzhou	119° 04'E, 29° 61'N
Honey-622	*Apis cerana*	2018	Mutiflower	apiary	Hangzhou	119° 04'E, 29° 61'N
Honey-623	*Apis cerana*	2018	Mutiflower	apiary	Hangzhou	119° 04'E, 29° 61'N
Honey-624	*Apis cerana*	2018	Mutiflower	apiary	Hangzhou	119° 04'E, 29° 61'N
Honey-625	*Apis mellifera*	2018	Longan	apiary	Hangzhou	119° 69'E, 29° 80'N
Honey-626	*Apis mellifera*	2018	Common Eurya	apiary	Hangzhou	119° 69'E, 29° 80'N
Honey-627	*Apis cerana*	2018	Mutiflower	apiary	Hangzhou	119° 69'E, 29° 80'N
Honey-628	*Apis cerana*	2018	Mutiflower	apiary	Hangzhou	119° 69'E, 29° 80'N
Honey-629	*Apis cerana*	2018	Chinese Magnoliavine Fruit	apiary	Hangzhou	119° 69'E, 29° 80'N
Honey-630	*Apis cerana*	2018	Mutiflower	apiary	Hangzhou	119° 69'E, 29° 80'N
Honey-631	*Apis mellifera*	2018	Longan	apiary	Jinhua	119° 46'E, 29° 21'N
Honey-632	*Apis mellifera*	2018	Mutiflower	apiary	Jinhua	119° 46'E, 29° 21'N
Honey-633	*Apis mellifera*	2018	Mutiflower	apiary	Jinhua	119° 46'E, 29° 21'N
Honey-634	*Apis mellifera*	2018	Longan	apiary	Jinhua	119° 46'E, 29° 21'N

Honey-635	*Apis mellifera*	2018	Mutiflower	apiary	Jinhua	120° 45'E, 29° 06'N
Honey-636	*Apis mellifera*	2018	Jujube	apiary	Jinhua	120° 07'E, 29° 31'N
Honey-637	*Apis mellifera*	2018	Mutiflower	apiary	Jinhua	120° 07'E, 29° 31'N
Honey-638	*Apis mellifera*	2018	Mutiflower	apiary	Huzhou	119° 91'E, 30° 01'N
Honey-639	*Apis mellifera*	2018	Mutiflower	apiary	Huzhou	119° 91'E, 30° 01'N
Honey-640	*Apis mellifera*	2018	Linden	apiary	Huzhou	119° 91'E, 30° 01'N
Honey-641	*Apis mellifera*	2018	Mutiflower	apiary	Huzhou	119° 91'E, 30° 01'N
Honey-642	*Apis mellifera*	2018	Mutiflower	apiary	Huzhou	119° 91'E, 30° 01'N
Honey-643	*Apis mellifera*	2018	Mutiflower	apiary	Huzhou	119° 91'E, 30° 01'N
Honey-644	*Apis mellifera*	2018	Mutiflower	apiary	Huzhou	119° 91'E, 30° 01'N
Honey-645	*Apis cerana*	2018	Mutiflower	apiary	Huzhou	119° 91'E, 30° 01'N
Honey-646	*Apis mellifera*	2018	Acacia	apiary	Huzhou	119° 91'E, 30° 01'N
Honey-647	*Apis mellifera*	2018	Mutiflower	apiary	Huzhou	119° 91'E, 30° 01'N
Honey-648	*Apis mellifera*	2018	Mutiflower	apiary	Shaoxing	120° 24'E, 29° 71'N
Honey-649	*Apis mellifera*	2018	Mutiflower	apiary	Shaoxing	120° 24'E, 29° 71'N
Honey-650	*Apis mellifera*	2018	Mutiflower	apiary	Shaoxing	120° 24'E, 29° 71'N
Honey-651	*Apis mellifera*	2018	Citrus	apiary	Taizhou	121° 44'E, 28° 67'N
Honey-652	*Apis mellifera*	2018	Longan	apiary	Taizhou	121° 44'E, 28° 67'N
Honey-653	*Apis mellifera*	2018	Acacia	apiary	Taizhou	121° 44'E, 28° 67'N
Honey-654	*Apis mellifera*	2018	Acacia	apiary	Taizhou	121° 39'E, 28° 37'N
Honey-655	*Apis mellifera*	2018	Mutiflower	apiary	Taizhou	121° 39'E, 28° 37'N
Honey-656	*Apis cerana*	2018	Mutiflower	apiary	Wenzhou	119° 72'E, 27° 56'N

Honey-657	*Apis cerana*	2018	Mutiflower	apiary	Wenzhou	120° 43'E, 27° 52'N
Honey-658	*Apis cerana*	2018	Mutiflower	apiary	Wenzhou	120° 09'E, 27° 79'N
Honey-659	*Apis cerana*	2018	Mutiflower	apiary	Wenzhou	120° 09'E, 27° 79'N
Honey-660	*Apis cerana*	2018	Mutiflower	apiary	Wenzhou	120° 09'E, 27° 79'N
Honey-661	*Apis cerana*	2018	Mutiflower	apiary	Wenzhou	120° 09'E, 27° 79'N
Honey-662	*Apis mellifera*	2018	Mutiflower	apiary	Quzhou	118° 42'E, 27° 14'N
Honey-663	*Apis cerana*	2018	Mutiflower	apiary	Quzhou	118° 42'E, 27° 14'N
Honey-664	*Apis cerana*	2018	Mutiflower	apiary	Quzhou	118° 42'E, 27° 14'N
Honey-665	*Apis cerana*	2018	Mutiflower	apiary	Quzhou	118° 42'E, 27° 14'N
Honey-666	*Apis cerana*	2018	Mutiflower	apiary	Quzhou	118° 42'E, 27° 14'N
Honey-667	*Apis cerana*	2018	Mutiflower	apiary	Quzhou	118° 63'E, 28° 74'N
Honey-668	*Apis cerana*	2018	Mutiflower	apiary	Quzhou	118° 63'E, 28° 74'N
Honey-669	*Apis cerana*	2018	Mutiflower	apiary	Quzhou	118° 63'E, 28° 74'N
Honey-670	*Apis mellifera*	2018	Longan	apiary	Quzhou	118° 63'E, 28° 74'N
Honey-671	*Apis cerana*	2018	Mutiflower	apiary	Quzhou	118° 63'E, 28° 74'N
Honey-672	*Apis cerana*	2018	Mutiflower	apiary	Lishui	119° 57'E, 28° 12'N
Honey-673	*Apis cerana*	2018	Mutiflower	apiary	Lishui	119° 57'E, 28° 12'N
Honey-674	*Apis cerana*	2018	Mutiflower	apiary	Lishui	119° 64'E, 27° 97'N
Honey-675	*Apis cerana*	2018	Mutiflower	apiary	Lishui	119° 64'E, 27° 97'N
Honey-676	*Apis cerana*	2018	Mutiflower	apiary	Lishui	119° 91'E, 28° 45'N
Honey-677	*Apis cerana*	2018	Mutiflower	apiary	Lishui	119° 91'E, 28° 45'N
Honey-678	*Apis cerana*	2018	Mutiflower	apiary	Lishui	119° 91'E, 28° 45'N

Honey-679	*Apis cerana*	2018	Mutiflower	apiary	Lishui	120° 09'E, 28° 66'N
Honey-680	*Apis cerana*	2018	Mutiflower	apiary	Lishui	120° 09'E, 28° 66'N
Honey-681	*Apis cerana*	2018	Mutiflower	apiary	Lishui	120° 09'E, 28° 66'N

Table S3 The MRM (Multiple reaction monitoring) transition parameters for FQs.

Compound	Precursor ion （m/z）	Product ions (m/z)	Declustering (eV)	Collision (eV)	Retention time (min)
Ciprofloxacin	332.0	288.0[a]	80	25	8.17
		245.0	80	33	
Danofloxacin	358.0	340.0[a]	80	30	5.41
		283.0	80	35	
Difloxacin	400.0	356.0[a]	80	28	5.72
		299.0	80	42	
Enoxacin	321.0	303.0[a]	80	35	4.70
		232.0	80	48	
Enrofloxacin	360.0	342.0[a]	80	30	8.27
		316.0	80	40	
Fleroxacin	370.0	326.0[a]	80	30	4.38
		269.0	80	40	
Flumequine	262.0	244.0[a]	80	30	7.13
		202.0	80	49	
Lomefloxacin	352.0	265.0[a]	80	33	5.56
		308.0	80	28	
Marbofloxacin	363.0	345.0[a]	80	25	4.28
		320.0	80	21	
Norfloxacin	320.0	305.0[a]	80	30	4.87
		276.0	80	26	
Ofloxacin	362.0	318.0[a]	80	30	4.66
		261.0	80	40	
Pefloxacin	334.0	316.0[a]	80	27	4.68
		290.0	80	25	
Sarafloxacin	386.0	342.0[a]	80	28	5.86
		299.0	80	43	

[a] The transition ion pair used for quantitation

Table S4 Exposure parameters used to perform probabilistic risk assessments of human exposure to FQs in honey.

Exposure parameter	Mean	SD
concentration of FQs in honey (C_j, μg/kg)		
Ciprofloxacin	1.06	10.88
Enrofloxacin	0.24	0.091
Norfloxacin	16.10	307.76
Ofloxacin	0.091	0.422
body weight (BW_j, kg)		
4-11	26.2	5.1
12-18	42.2	7.9
19-64	62.5	11.2
≥65	62.6	10.8
Honey consumption (K_j, kg·day^{-1})		
4-11	0.0015	0.0002
12-18	0.0032	0.0003
19-64	0.0101	0.0010
≥65	0.0093	0.0009

Table S5 The results of FQ residues in honey from different regions Zhejiang Province, China.

**Region**	**Number of positive samples**	**Number of total samples**	**Detection rate (%)**	**Average residue (μg/kg)**
Hangzhou	3	104	2.9	127.0 ± 204.4
Huzhou	6	51	11.8	56.7 ± 64.0
Jiaxing	1	20	5.0	2.3
Jinhua	4	118	3.4	30.9 ± 30.5
Lishui	4	123	3.3	57.8 ± 58.1
Ningbo	13	74	17.6	41.4 ± 49.9
Quzhou	13	150	8.7	757.7 ± 2180.5
Shaoxing	3	30	10.0	11.3 ± 14.0
Taizhou	0	5	0.0	0.0
Wenzhou	0	6	0.0	0.0

Table S6 The results of FQ residues in honey from different Years in Zhejiang Province, China.

Year	Number of positive samples	Number of total samples	Detection rate (%)	Average residue (μg/kg)
2014	5	82	6.1	354.2 ± 638.3
2015	2	153	1.3	15.5 ± 18.2
2016	15	115	13.0	53.2 ± 54.1
2017	11	189	5.8	773.0 ± 2362.7
2018	14	142	9.9	26.8 ± 42.2
